# FGF15/FGFR4 signaling suppresses M1 macrophage polarization and multi-organ inflammation in septic mice by inhibiting H3K18 lactylation-driven *Irf7* expression through NF2-Hippo activation

**DOI:** 10.1038/s41419-025-07962-w

**Published:** 2025-08-19

**Authors:** Bo Li, Jiayu Li, Zexiang Zhu, Yong Tang, Yun Zhou, Geshu Du, Xing Li

**Affiliations:** 1 Department of Critical Care Medicine, Changsha Hospital of Traditional Chinese Medicine (Changsha No. 8 Hospital), Changsha City, Hunan Province PR China; 2 Department of Acupuncture, Moxibustion & rehabilitation, Changsha Hospital of Traditional Chinese Medicine (Changsha No. 8 Hospital), Changsha City, Hunan Province PR China

**Keywords:** Inflammasome, Sepsis, Drug delivery

## Abstract

M1 macrophage polarization plays a key role in the onset and progression of sepsis. Fibroblast growth factor 15 (FGF15) suppresses septic inflammation through its FGF receptor 4 (FGFR4); however, the underlying mechanisms are largely unclear. In this study, we evaluated the anti-inflammatory effects of recombinant FGF15 (rFGF15) in cecal ligation and puncture (CLP)-induced septic mice in vivo, as well as lipopolysaccharide (LPS)-stimulated mouse bone marrow-derived macrophages (BMDMs) and RAW264.7 macrophages in vitro. We observed that rFGF15 suppressed M1 macrophage polarization and associated inflammatory responses in both CLP-induced septic mice and LPS-stimulated BMDMs and RAW264.7 macrophages. Additionally, macrophage-depleted CLP mice transplanted with LPS-stimulated BMDMs pre-treated with rFGF15 exhibited reduced multi-organ inflammation and enhanced survival compared to those receiving LPS-stimulated BMDMs without rFGF15 treatment. Mechanistically, FGF15 activated the neurofibromin 2 (NF2)-Hippo pathway through FGFR4, leading to the inhibition of glycolysis, lactate production, and histone H3K18 lactylation. This led to reduced expression of interferon regulatory factor 7 (Irf7), a key regulator of type I interferon responses. In conclusion, FGF15 suppresses M1 macrophage polarization and associated inflammatory responses in sepsis by activating the NF2-Hippo pathway, thereby inhibiting H3K18 lactylation-driven *Irf7* expression. FGF15 holds promise as a potential innovative therapy for sepsis.

## Introduction

Sepsis is a life-threatening emergency that occurs when the human body’s immune response to an infection becomes overreactive and toxic, causing damages to vital organs, even death. In 2017 alone, there were 11 million sepsis-related deaths reported globally, accounting for roughly 20% of all deaths worldwide [1]. The management of sepsis involves three primary approaches: infection control, hemodynamic stabilization, and modulation of the septic immune response. Infection control is typically achieved through the timely administration of antibiotics, while hemodynamic stabilization is managed with fluid resuscitation and the use of vasoactive agents. However, no therapeutics targeting specific immune components of the septic response have consistently demonstrated improved outcomes in randomized clinical trials [2]. The most widely used intervention in this category is the administration of glucocorticoids, which has a significant but rather limited impact on septic response and mortality. The discovery and development of novel therapeutics directed at specific components of the septic response are essential to reducing patient morbidity and mortality.

Macrophages serve as the first line of defense in the innate immune system, primarily through their role as phagocytes, where they engulf and eliminate invading pathogens. They also secret inflammatory cytokines to recruit additional immune cells to the site of infection and present antigens to activate adaptive immune responses. Macrophages exhibit high heterogeneity and plasticity, allowing them to polarize into proinflammatory M1-like and anti-inflammatory M2-like phenotypes in response to various stimuli [3]. In the early stages of sepsis, a predominant M1 macrophage response drives excessive inflammation and tissue damage [4]. Researchers are evaluating the therapeutic potential of modulating macrophage polarization, with a focus on suppressing excessive M1 polarization, as an innovative treatment for sepsis [4].

Recent metabolic studies have revealed that macrophage polarization is tightly associated with cellular metabolic reprogramming [5]. In sepsis, glycolysis supersedes oxidative phosphorylation (OXPHOS) as the primary energy-producing pathway, driving excessive M1 macrophage polarization and contributing to uncontrolled septic inflammation and organ damage [6, 7]. Histone lactylation, a newly discovered epigenetic modification that relies on lactate, a byproduct of glycolysis, influences macrophage activation and polarization [8, 9]. Histone lactylation is widely recognized as a crucial regulator of sepsis pathogenesis. Specifically, lactylated histone H3 lysine 18 (H3K18la) was markedly elevated in patients with septic shock and its levels were significantly correlated with disease severity and prognosis [10]. H3K14la enrichment at promoter regions of ferroptosis-related genes drives sepsis-associated pulmonary endothelial cell ferroptosis [11]. This study was the first report on the role of H3K18la in regulating sepsis-related M1 macrophage polarization.

Fibroblast growth factor 19 (FGF19) and its rodent ortholog FGF15 are hormone-like proteins secreted primarily by ileal epithelial cells in response to bile acid stimulation. By specifically activating the FGF receptor 4 (FGFR4), they regulate hepatic glucose metabolism through the intestine-liver crosstalk [12]. FGF15 has been shown to activate Hippo, a signaling pathway closely integrated with glucose metabolism [13], through neurofibromin 2 (NF2/Merlin) [14]. Intriguingly, the FGF15/FGFR4 signaling has been implicated in the pathogenesis of inflammatory bowel disease by regulating macrophage polarization [15]. In 2022, Li and colleagues reported that FGF15 markedly reduced hepatic inflammation in mice with cecal ligation and puncture (CLP)-induced sepsis and significantly prolonged their survival [16]; however, the underlying mechanisms remain largely unclear. In the present study, we evaluated the effects of recombinant FGF15 (rFGF15) on macrophage polarization and multi-organ inflammation in mice with CLP-induced sepsis. The underlying mechanisms, including the NF2-Hippo signaling pathway, glycolysis, lactate production, and histone lactylation, were investigated. The findings from this study supported FGF15 as a potential therapeutic intervention for critically ill patients.

## Methods

### Animals

Male C57BL/6 mice (7–8-week old, weighing 18–22 g) were purchased from Hunan Slake Jingda Experimental Animal Co., Ltd. (Changsha, China). The animals were acclimated for one week in a specific pathogen-free (SPF) environment, maintained on a 12 h light/dark cycle, with free access to water and food. All animal protocols were approved by the Changsha Hospital of Traditional Chinese Medicine (Changsha No. 8 Hospital) committee (the approval number:2023110804) and followed the ARRIVE guidelines (Animal Research: Reporting of In Vivo Experiments) and the Guide for the Care and Use of Laboratory Animals.

### Mouse macrophage isolation

The C57BL/6 mice were euthanized with intraperitoneal sodium pentobarbital (120 mg/kg). The lungs, livers, intestines, kidneys, femurs, and tibias were collected. For the isolation of tissue-derived macrophages, the lung, liver, intestine, and kidney tissues were digested with collagenase and/or trypsin, and the homogenates were filtered through a 100 μm membrane and centrifuged at 1000 rpm/min for 5 min. Macrophages were isolated from the homogenates using Anti-F4/80 MicroBeads (130-110-443, Miltenyi Biotech, Cologne, Germany) according to the manufacturer’s instructions. For the isolation of peritoneal macrophages, the peritoneal cavity was repeatedly washed with RPMI 1640 medium, and the combined lavage fluid was filtered through a 100 μm membrane and centrifuged at 1000 rpm/min for 5 min. Macrophage colony-stimulating factor (M-CSF) (20 ng/mL) was added to induce macrophage differentiation for 7 days. For the isolation of bone marrow-derived macrophages (BMDMs), bone marrow cells were harvested from the femoral and tibial cavities by repeatedly flushing them with RPMI 1640 medium. The combined lavage fluid was filtered through a 100 μm membrane and centrifuged at 1000 rpm/min for 5 min. Following the removal of erythrocytes using red blood cell lysis buffer, the remaining cells were induced with 20 ng/mL M-CSF for 7 days to generate BMDMs.

### Cell culture and interventions

Mouse BMDMs isolated and induced as above were cultured in RPMI 1640 medium supplemented with 10% fetal bovine serum (FBS) and 1% penicillin-streptomycin (Pen-Strep). RAW264.7 mouse macrophages (AW-CCM002, Abiowell, Changsha, China) were maintained in Dulbecco’s modified Eagle’s medium (DMEM) containing 10% FBS and 1% Pen-Strep. RAW264.7 cells were authenticated through short tandem repeat (STR) profiling and tested for mycoplasma contamination. Both BMDMs and RAW264.7 cells were maintained at 37 °C in a humidified atmosphere containing 5% CO_2_.

All cells were randomly assigned to receive different drug interventions. Specifically, following stimulation with 100 μg/mL lipopolysaccharide (LPS) for 24 h, the cells were incubated with 200 ng/mL recombinant FGF15 protein (rFGF15; CSB–EP522052MO, Cusabio, Wuhan, China) for 24 h as previously described [16]. To explore the functional roles of FGFR4, NF2, and interferon regulatory factor 7 (Irf7), the cells were transfected with FGFR4-silencing plasmids (si-FGFR4; HG-SM008011, HonorGene, Changsha, China), NF2-silencing plasmids (si-NF2; HG-SM010898, HonorGene), and Irf7-expressing plasmids (oe-Irf7; HG-MO016850, HonorGene), respectively. The cells transfected with empty vector (si-NC or oe-NC) served as negative control. To explore the functional role of NF2 phosphorylation, the cells were transfected with plasmids carrying wild-type NF2 (NF2-WT; HG-MO252250, HonorGene) or phospho-null (Y207F) NF2 mutant (NF2-Mut; HG-MO252250-Y207F, HonorGene). All cell transfections were conducted using lipofectamine 2000 according to the manufacturer’s instructions. The sequences of si-FGFR4, si-NF#1, and si-NF#2 were GGCCAGATACACAGATATCAT, GAGTTCAACTGCGAGATGAAA, and GGGTGATAAATCTCTATCAGA, respectively. To explore the functional role of NF2 in repair phenotype, the cells were stimulated with 10 ng/mL interleukin-4 (IL-4) and 10 ng/mL IL-13 for 24 h [17], followed by incubation with 200 ng/mL rFGF15 protein.

### Mouse model of sepsis

C57BL/6 mice were randomly subjected to cecal ligation and puncture (CLP) to induce a condition mimicking human sepsis as previously described [10]. In brief, following anesthetization with 50 mg/kg sodium pentobarbital, a 1-cm midline incision was made in the abdomen to expose the cecum. The cecum was ligated midway between its distal pole and base and punctured twice near the distal end with a 20-gauge needle. A small amount of fecal matter was gently extruded, after which the cecum was repositioned, and the abdominal muscle layer and skin were sutured. Sham-operated mice were subjected to the same surgical procedure, excluding the CLP. After resting for 2 h, CLP mice randomly received tail vein injection of 400 μg/kg rFGF15 or saline alone every 12 h for 3 days [16]. The sham, CLP, and CLP + rFGF15 groups each contained 10 mice. The sample size was calculated using efficacy analyses [18]. The mice were sacrificed 12 h after the final rFGF15 injection using intraperitoneal administration of sodium pentobarbital (120 mg/kg). Tissue samples and peripheral blood were collected for molecular and histological analysis.

### Macrophage depletion and transplantation in vivo

Four days before the CLP surgery, liposomal clodronate (200 μL) was intravenously administered once daily to deplete macrophages [19]. BMDMs were collected and analyzed with flow cytometry pre- and post-CLP. For BMDM transplantation, 3 × 10^6^ BMDMs from wild-type donor mice were pre-treated with 100 ng/mL LPS or 200 ng/mL rFGF15 for 24 h, or transfected with oe-Irf7 or oe-NC for 24 h, alone or in combination as specified. The cells were then transplanted into macrophage-depleted recipient mice via tail vein injection immediately before the CLP surgery. The sham, CLP, CLP + BMDM, CLP + BMDM^LPS^, CLP + BMDM^LPS+rFGF15^, CLP + BMDM ^LPS+rFGF15+oe-NC^, and CLP + BMDM ^LPS+rFGF15+oe-Irf7^ groups each contained 20 mice. Survival was assessed every 24 h over a 7-day period. For molecular and histological analysis, the mice were sacrificed using intraperitoneal administration of sodium pentobarbital (120 mg/kg) 72 h after CLP.

### Histological analysis

Fresh heart, liver, intestine, lung, and kidney tissues were fixed in 4% paraformaldehyde, embedded in paraffin, and cut into 4 µm sections. The sections were stained with hematoxylin (AWI0001a, Abiowell) and eosin (AWI0029a, Abiowell), and tissue damage was assessed under an optical microscope (BA210T, Motic, Xiamen, China).

### Lung edema assessment

Fresh lung specimens were weighed to obtain wet weight. The specimens were subsequently dehydrated in an oven at 60 °C for 72 h and weighed to obtain dry weight. The wet/dry ratios were computed to reflect pulmonary edema.

### Assessment of bacterial loads

As previously described [20], serial dilutions of mouse peripheral blood and peritoneal lavage fluid (PLF) were cultured on soybean-casein digest agar medium (146004, Millipore, Billerica, MA, USA) at 37 °C overnight, followed by bacterial colony-forming unit (CFU) counting.

### Flow cytometry

The cells were fixed and permeabilized using intracellular fixation and permeabilization buffer (eBioscience, San Diego, CA, USA), stained with antibodies toward target proteins according to the manufacturer’s instructions, and subjected to flow cytometric analysis on a flow cytometer (A00-1-1102, Beckman Coulter, Fullerton, CA, USA). The antibodies used were as follows (all from eBioscience): CD11b-FITC (0.5 µg/test; 11-0112-82), F4/80-APC (0.25 µg/test;12-4801-82), CD45-PC7 (25-0451-82), CD86-APC (0.06 µg/test; 17-0862-82), CD206-APC (0.25 µg/test; 17-2061-82), CD4-FITC (0.25 µg/test; 11-0041-82), IL-17A-PE (0.125 µg/test; 12-7177-81), CD25-APC (0.125 µg/test; 17-0251-82), and forkhead box protein P3 (Foxp3)-PE (1 µg/test; 12-5773-82). The M1/M2 and T-helper 17 (Th17)/T-regulatory (Treg) cell ratios were determined using the FlowJo software (TreeStar, Ashland, OR, USA). BMDMs were characterized by the expression of CD11b, CD45, and F4/80.

### Western blot analysis

The mouse tissues and macrophages were lysed using RIPA lysis buffer (R0010, Solarbio, Beijing, China). The protein concentrations were determined using a bicinchoninic acid (BCA) kit. The proteins were separated using sodium dodecyl sulphate (SDS)-polyacrylamide gel electrophoresis and transferred to nitrocellulose membranes. After blocking with TBST containing 5% skim milk, the membranes were incubated with primary antibodies, followed by appropriate HRP-conjugated secondary antibodies. Protein bands were visualized using an enhanced chemiluminescence (ECL) reagent and detected on a ChemiScope6100 system (CLiNX, Shanghai, China). The protein expression levels were quantified using ImageJ software (NIH, Bethesda, MD, USA) and normalized to β-actin. The antibodies used are presented in Supplementary Table 1.

### Enzyme-linked immunosorbent assay (ELISA)

Mouse serum was collected by centrifugation of peripheral blood (1000 g, 15 min) at 4 °C and stored at −80 °C. Cell lysates, culture medium, and serum samples were subjected to ELISA using the following commercial kits according to the manufacturer’s instructions: TNF-α (KE10002, Proteintech, Rosemont, IL, USA), IL-1β (KE10003, Proteintech), IL-6 (KE10007, Proteintech), COX-2 (CSB-E12910m, Cusabio), iNOS (CSB-E08326m, Cusabio), IL-10 (KE10008, Proteintech), TGF-β (KE10005, Proteintech), BNP (EM0877, Wuhan Fine Biotech Co., Ltd., Wuhan, China), CK-MB (EM0929), and cTnI (JL11280, Jianglai Industry Co., Ltd., Shanghai, China).

### High Performance liquid chromatography-tandem mass spectrometry (HPLC-MS/MS)

The levels of glucose 6-phosphate (G6P), fructose 6-phosphate (F6P), 3-phosphoglycerate (3PG), 2-phosphoglycerate (2PG), phosphoenolpyruvate (PEP), acetyl coenzyme A (acetyl-CoA), adenosine diphosphate (ADP), and adenosine triphosphate (ATP) were determined using HPLC-MS/MS in mouse BMDMs treated with vehicle (Control), LPS, or LPS + rFGF15. The analysis was conducted on a QTRAP 5500 mass spectrometer (SCIEX) coupled to a ExionLC AD UPLC system (SCIEX), which was equipped with a ZIC-pHILIC column (5 μm, 2.1 × 100 mm, PN: 1.50462.0001, Millipore) maintained at 40 °C. The loading volume was 3 μL for each sample. The mobile phases consisted of 25 mM ammonium acetate in 25 mM ammonia hydroxide (mobile phase A) and acetonitrile (90/10, v/v) (mobile phase B). The gradient program was as follows: 95% B for 1 min, decreased to 65% B over 14 min, further decreased to 40% B over 16 min and held for 18 min, increased back to 95% B over 18 min, and finally reduced back to 9% B and held for another 23 min. The flow rate was 0.3 mL/min. The mass spectra were acquired using a Turbo V ion source operating in negative mode, with a spray voltage of −4500 V, a source temperature of 550 °C, a gas 1 pressure of 50 psi, a gas 2 pressure of 55 psi, and a curtain gas pressure of 40 psi. Metabolite peaks were detected in single ion monitoring mode, with delustering potentials and collision energies optimized using analytical standards. The m/z for the metabolites were as follows: 259.0/96.9 for G6P, 259.0/97.0 for F6P, 184.9/96.9 for 3PG/2PG, 166.9/78.9 for PEP, 808.0/408.0 for acetyl-CoA, 425.9/133.9 for ADP, and 505.9/408.0 for ATP. Data were collected using the Analyst 1.7.1 software (SCIEX). The relative amounts of metabolites were calculated using the MultiQuant 3.0.3 software (SCIEX).

Protein extracts from mouse BMDMs treated with vehicle (Control) or LPS were subjected to tryptic digestion. Lactylated peptides were enriched following using a lactylation-specific antibody resin enrichment kit. Total peptides from each sample were subjected to analysis on a nano-UPLC system (Vanquishneo) coupled to a mass spectrometer (Astral) equipped with a nano-electrospray ion source. Chromatographic separation was performed on a 150 μm ID × 15 cm reversed-phase column (EASY-Spray™ HPLC, Thermo Scientific). The mobile phase consisted of acetonitrile-water-formic acid, with phase A containing 0.1% formic acid in water and phase B containing 0.1% formic acid in 80% acetonitrile. Mass spectrometry analysis was performed using data-independent acquisition (DIA) in positive ion mode. Full-scan MS1 spectra were acquired over a range of 380–980 m/z at a resolution of 240,000, with an isolation window of 2 m/z and normalized collision energy (NCE) of 25%. MS2 spectra were collected from 150 to 2000 m/z using absolute AGC values of 5E4 and maximum injection times of 3 ms. Raw spectral data were processed using the Spectronaut software (version 19.0.240604.62635; Biognosys AG).

### CUT&Tag assays

CUT&Tag analysis was performed on mouse BMDMs treated with LPS or LPS + rFGF15. Cells were immobilized using concanavalin A-coated magnetic beads and permeabilized with the nonionic detergent digitonin. The cells were subsequently incubated with anti-H3K18la antibody (1:100; PTM-1427RM, PTM BIO) followed by secondary antibody. Protein A/G-Tn5 (pA/G-Tn5) was introduced to liberate antibody-bound DNA fragments from chromatin into extracellular space. DNA was extracted and fragments below 700 bp were isolated via magnetic bead-based size selection. PCR amplification was conducted followed by quality verification using 1% agarose gel electrophoresis. Sequencing libraries were subjected to high-throughput sequencing. Raw data underwent quality control through the fastp software (version 0.23.1). Clean reads were aligned to the reference genome (Ensembl_GRCm39_111). Broad peak scanning was implemented using MACS2 to identify chromosomal peak distributions. This process ultimately confirmed H3K18la enrichment at the *Irf7* loci.

### Biochemical assays

Lactate concentrations in the culture medium were determined using a lactic acid test kit (A019-2-1, Nanjing Jiancheng Bioengineering Institute, Nanjing, China). Glucose concentrations in cell lysates were determined using a glucose uptake colorimetric assay kit (MAK083, Sigma-Aldrich, St. Louis, MO, USA). Serum samples were subjected to functional marker detection using the following commercial kits. Scr (C011-2-1), BUN (C013-2-1), ALT (C009-2-1), TB (C019-1-1), ALB (A028-2-1) were purchased from Nanjing Jiancheng Bioengineering Institute; PT (BC8081) was purchased from Solarbio (Beijing, China). The assays were conducted following the manufacturer’s instructions.

### Co-immunoprecipitation (Co-IP)

The cells were lysed with IP lysis buffer. To explore the interaction between FGFR4 and NF2, the cell lysates were incubated with anti-FGFR4 antibody (1:50; 8562S, CST, Danvers, MA, USA) or IgG (B900610, Proteintech) overnight at 4 °C, followed by Protein A/G agarose beads for 4 h at 4 °C. The agarose beads were washed thoroughly with IP lysis buffer to remove non-specific proteins, and the resulting immunoprecipitated protein complexes were subjected to western blot analysis.

### RNA sequencing (RNA-seq)

RNA-seq and data analysis were conducted by Shanghai OE Biotech Co., Ltd. RNA-seq was performed on BMDMs treated with vehicle (Control), LPS, or LPS + rFGF15. Three independent samples were analyzed for each group. Sequence alignment was performed using HISAT2 software, while read counts were registered with HTSeq-count. DESeq2 software was used for differential analysis and visualization of gene read counts for each sample. The raw data can be found in NCBI (https://www.ncbi.nlm.nih.gov/bioproject/PRJNA1281990).

### Quantitative real-time PCR (qRT-PCR)

Total RNA was isolated using Trizol reagent (15596026, Thermo Scientific, Portsmouth, NH, USA). Following quantification on a Micro Drop ultra microspectrophotometer (BIO-DL, Shanghai, China), the RNA was reverse transcribed into cDNA using the HiFiScript cDNA Synthesis Kit (CW2569M, CWBIO, Taizhou, China). The qRT-PCR was performed using UltraSYBR Mixture (CW2601M, CoWin Biosciences) on a QuantStudio1 Real-Time PCR System (Thermo Scientific). The primers used for amplification are listed in Supplementary Table 2. The mRNA expression levels were determined using the 2^−ΔΔCt^ method and normalized to *β-actin*.

### Chromatin immunoprecipitation (ChIP)

The enrichment of H3K18la at the promoter regions of *Irf7*, *Ndrg1*, *Odc1*, and *Rsad2* was analyzed by ChIP in combination with qRT-PCR. In brief, 3 × 10^6^ cells were treated with 1.1% formaldehyde to crosslink chromatin proteins to DNA, and the crosslinking reaction was terminated by the addition of glycine. To isolate genomic DNA, the cells were resuspended in lysis buffer, and the chromatin was fragmented by sonication. The size of the DNA fragments was assessed using a 1.5% agarose gel. The cell lysates containing fragmented DNA with crosslinked chromatin proteins were incubated with anti-lgG (6 μg/5 × 10^6^ cells; 30000-0-AP, Proteintech), anti-H3K18la (6 μg/5 × 10^6^ cells; ab40888, Abcam, Cambridge, UK), or anti-H3 antibody (6 μg/5 × 10^6^ cells; ab1791, Abcam). The immunoprecipitated DNA-protein complexes were treated with proteinase K, and the purified DNA fragments were analyzed by qRT-PCR using specific primers for the *Irf7*, *Ndrg1*, *Odc1*, and *Rsad2* promoters (Supplementary Table 2).

### Statistical analysis

Data are presented as mean ± standard deviation (SD). Statistical analysis was performed using GraphPad Prism software (version 9). Normality of distribution was assessed using the Shapiro-Wilk test. Comparisons among multiple groups were conducted using analysis of variance (ANOVA), followed by Tukey’s multiple comparisons. Survival was analyzed using the Kaplan-Meier method. All experiments were randomized and blindly analyzed to minimize experimental bias. A two-tailed *p* value of less than 0.05 was considered statistically significant.

## Results

### FGF15/FGFR4 signaling inhibits glycolysis in septic macrophages

**Fig. 1:**
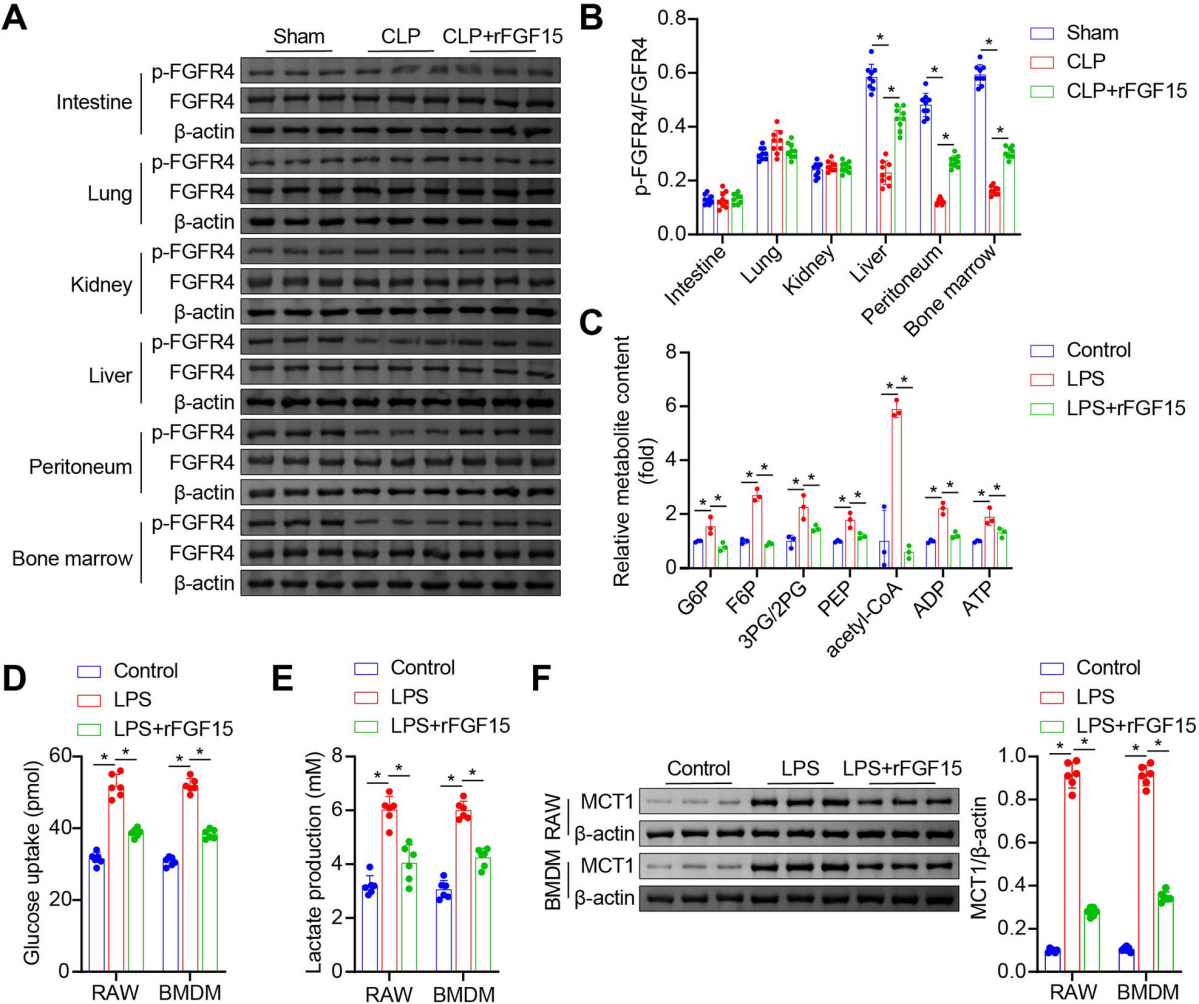
FGF15/FGFR4 signaling inhibits glycolysis in septic macrophage. **A**, **B** Detection of p-FGFR4 and FGFR4 in macrophages derived from the intestine, lung, kidney, liver, peritoneum, and bone marrow of Sham, CLP, and rFGF15-treated CLP (CLP+rFGF15) mice by western blot analysis. **A** Immunoblot images. **B** Quantified p-FGFR4/FGFR4 ratios. *n* = 9, *p < 0.05. **C** Detection of glycolytic metabolites in control, LPS-stimulated (LPS), and rFGF15-treated, LPS-stimulated (LPS + rFGF15) mouse BMDMs by HPLC-MS/MS analysis. *n* = 3, *p < 0.05. **D**, **E** Glucose uptake (**D**) and lactate production (**E**) in control, LPS, and LPS + rFGF15 mouse BMDMs or RAW264.7 macrophages. **F** Detection of MCT1 protein expression in mouse BMDMs and RAW264.7 macrophages treated with control, LPS, or LPS + rFGF15 by western blot analysis. *n* = 6, *p < 0.05.

To explore the function of FGF5/FGFR4 signaling in septic macrophages, we collected macrophages from major organs, peritoneum, and bone marrow of CLP-induced septic and sham control mice. Western blot analysis revealed a significant downregulation of the phospho-FGFR4 (p-FGFR4)/FGFR4 ratio in macrophages derived from the liver, peritoneum, and bone marrow of CLP septic mice (Fig. 1A, B). The administration of rFGF15 through tail vein injection restored the p-FGFR4/FGFR4 ratio in CLP mice (Fig. 1A, B). HPLC-MS/MS analysis demonstrated a significant increase in glycolytic metabolites, including G6P, F6P, 3PG/2PG, PEP, acetyl-CoA, ADP, and ATP, in mouse BMDMs treated with LPS, indicating glycolytic upregulation. Treatment with rFGF15 normalized these glycolytic metabolites (Fig. 1C). In in vitro experiments, LPS stimulation of mouse BMDMs and RAW264.7 macrophages resulted in increased glucose uptake and lactate production (Fig. 1D, E), accompanied by upregulated protein levels of key glycolytic enzymes, including HK1, HK2, HK3, PFKM, PFKP, PFKL, PFKFB1, PFKFB2, PFKFB3, PFKFB4, PKM2, LDHA, LDHB, and LDHC (Fig. S1). Additionally, the expression of monocarboxylate transporter 1 (MCT1), a rate-limiting protein for lactate transmembrane transport [21], was elevated (Fig. 1F). Notably, LPS-induced upregulation of glucose uptake, lactate production, and glycolytic enzyme and MCT1 expression were all reversed by treatment with rFGF15 (Fig. 1D–F, Fig. S1). These in vivo and in vitro results suggested that FGF15/FGFR4 signaling inhibits glycolysis in septic macrophages.

### FGF15/FGFR4 signaling downregulates histone lactylation in septic macrophages

**Fig. 2:**
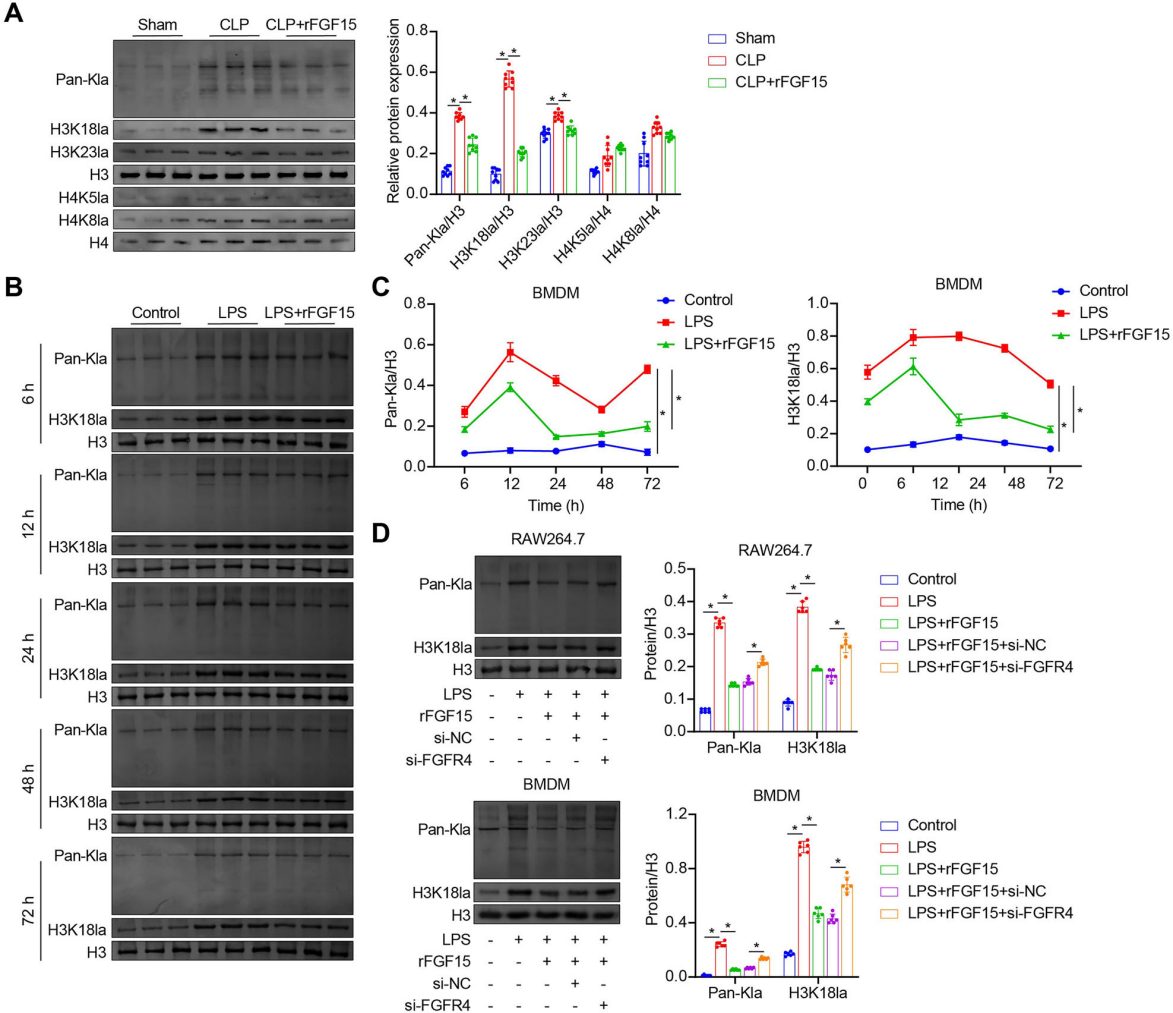
FGF15/FGFR4 signaling regulates histone lactylation in septic macrophages. **A** Detection of total lactylated histones (Pan-Kla), H3K18la, H3K23la, H3, H4K5la, H4K8la, and H4 in BMDMs of Sham, CLP, and rFGF15-treated CLP (CLP + rFGF15) mice by western blot analysis. *n* = 9, *p < 0.05. **B**, **C** Detection of Pan-Kla and H3K18la in mouse BMDMs following treatment with vehicle (Control), LPS, or LPS + rFGF15 for 6, 12, 24, 48, or 72 h by western blot analysis. **B** Immunoblot images. **C** Quantified Pan-Kla and H3K18la levels. **D** Detection of Pan-Kla and H3K18la in mouse BMDMs and RAW264.7 macrophages following treatment with vehicle (Control), LPS, LPS + rFGF15, LPS + rFGF15 + si-NC, or LPS + rFGF15 + si-FGFR4 for 48 h by western blot analysis. *n* = 6, *p < 0.05.

Histone lactylation is a newly discovered epigenetic modification that relies on lactate, a byproduct of glycolysis [8], and is associated with the severity and mortality of sepsis [10]. LC-MS/MS analysis identified a number of BMDM histone lactylations affected by LPS, including H4K59la, H2K99la, H4K31la, H1K16la, H2K5la, H3K56la, H1K33la, and H2K95la (downregulation), as well as H3K23la, H3K18la, H4K8la, and H4K5la (upregulation) (Fig. S2, Supplementary file 1). Having found that rFGF15 inhibited glycolysis and lactate production in LPS-stimulated mouse macrophages, we examined its effects on histone lactylation, including the lactylation of all histones and at specific histone lactylation sites including H3K23, H3K18, H4K8, and H4K5, in septic macrophages in vivo and in vitro. Western blot analysis revealed an increase in total lactylated histones, including H3K18la and H3K23la, in BMDMs from CLP-induced septic mice. Treatment with rFGF15 effectively reduced the levels of these lactylated proteins (Fig. 2A), with more pronounced changes observed for H3K18la in the treatment group. In in vitro experiments, LPS stimulation of mouse BMDMs and RAW264.7 macrophages resulted in increased levels of total lactylated histones and H3K18la. Treatment with rFGF15 reduced the levels of these lactylated proteins, an effect that was abolished by FGFR4 knockdown (Fig. 2B–D). These in vivo and in vitro results suggested that FGF15/FGFR4 signaling downregulates histone lactylation in septic macrophages.

### FGF15/FGFR4 signaling inhibits M1 polarization of septic macrophages and their inflammatory responses

**Fig. 3:**
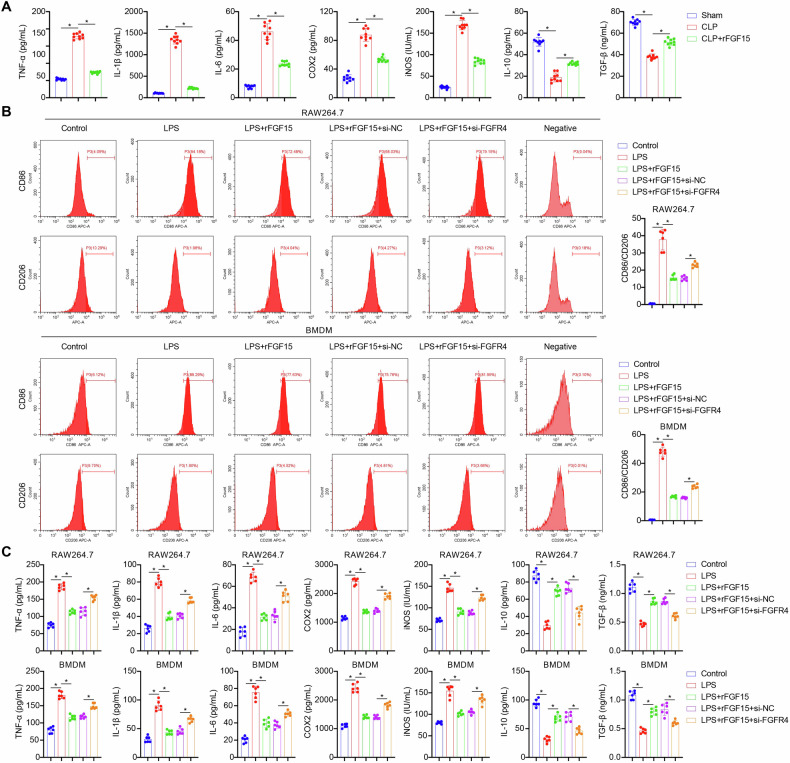
FGF15/FGFR4 signaling inhibits M1 polarization of septic macrophages and their inflammatory responses. **A** Detection of the pro-inflammatory mediators TNF-α, IL-1β, IL-6, COX-2, and iNOS and anti-inflammatory mediators IL-10 and TGF-β in peripheral blood of Sham, CLP and rFGF15-treated CLP (CLP + rFGF15) mice by ELISA. *n* = 9, *p < 0.05. **B** Detection of CD86 (a marker of M1-like macrophages) and CD206 (a marker of M2-like macrophages) in mouse BMDMs and RAW264.7 macrophages following treatment with vehicle (Control), LPS, LPS + rFGF15, LPS + rFGF15 + si-NC, or LPS + rFGF15 + si-FGFR4 by flow cytometry. **C** Detection of the pro-inflammatory mediators TNF-α, IL-1β, IL-6, COX-2, and iNOS and anti-inflammatory mediators IL-10 and TGF-β in mouse BMDMs and RAW264.7 macrophages following treatment with vehicle (Control), LPS, LPS + rFGF15, LPS + rFGF15 + si-NC, or LPS + rFGF15 + si-FGFR4 by ELISA. *n* = 6, *p < 0.05.

Next, we investigated the role of FGF15/FGFR4 signaling in septic macrophage polarization and inflammatory response. We detected elevated serum levels of the pro-inflammatory mediators TNF-α, IL-1β, IL-6, COX-2, and iNOS, along with decreased levels of the anti-inflammatory mediators IL-10 and TGF-β, in CLP-induced septic mice. These typical features of M1-associated inflammation in septic mice were attenuated by rFGF15 administration (Fig. 3A). In in vitro experiments, LPS stimulation of mouse BMDMs and RAW264.7 macrophages resulted in an increased ratio of CD86 + M1-like macrophages to CD206 + M2-like macrophages (Fig. 3B). This LPS-induced M1 polarization was associated with cellular inflammation, characterized by upregulated pro-inflammatory mediators and downregulated anti-inflammatory mediators (Fig. 3C). Notably, treatment with rFGF15 restored the M1/M2 ratio and mitigated cellular inflammation, effects that were reversed by FGFR4 knockdown (Fig. 3B, C). These in vivo and in vitro results suggested that FGF15/FGFR4 signaling inhibits M1 polarization of septic macrophages and their inflammatory responses.

### FGF15/FGFR4 signaling activates the NF2-Hippo pathway in septic macrophages

**Fig. 4:**
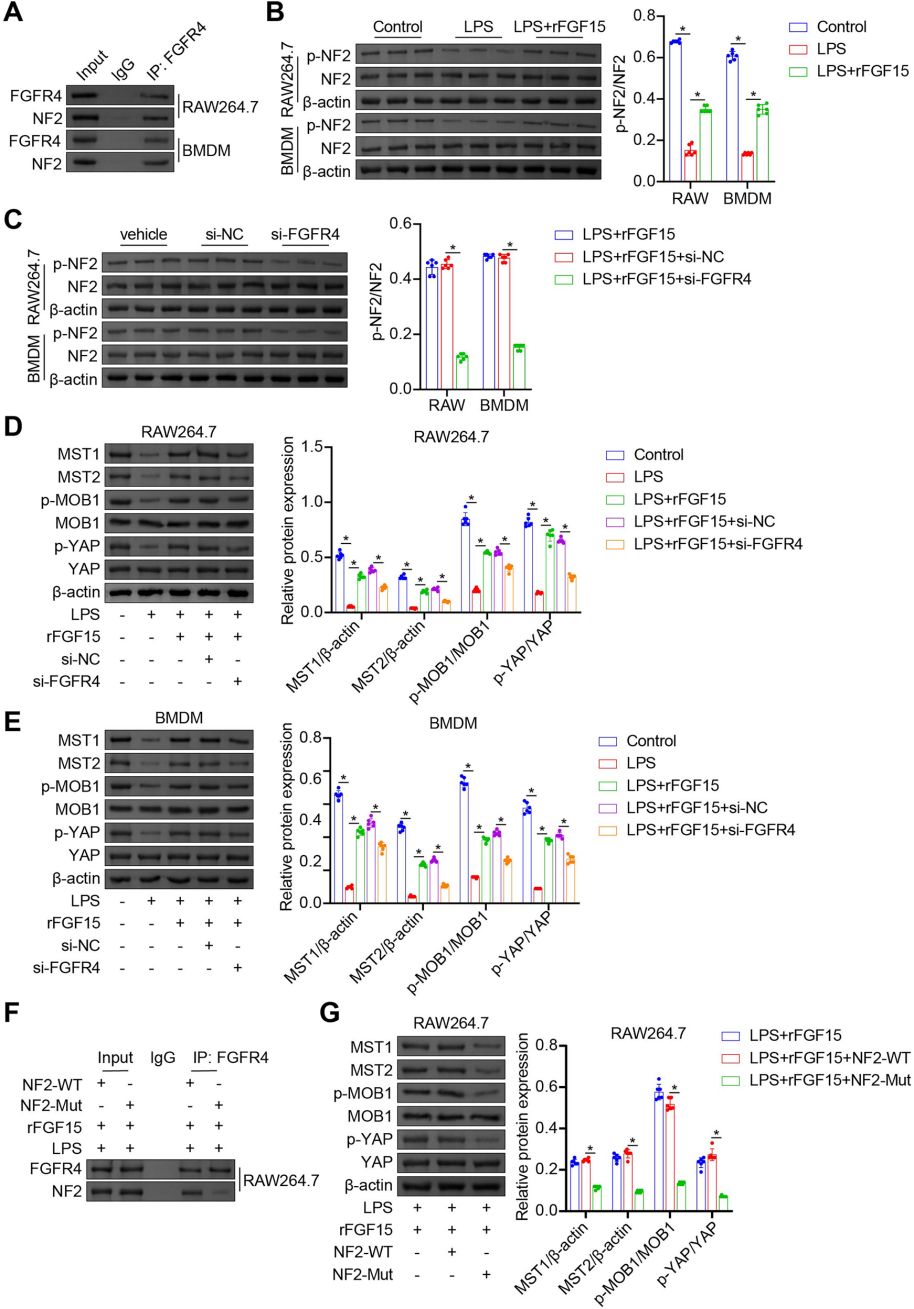
FGF15/FGFR4 signaling activates the NF2-Hippo pathway in septic macrophages. **A** Detection of the interaction between FGFR4 and NF2 in mouse BMDMs and RAW264.7 macrophages by Co-IP. **B**, **C** Detection of p-NF2 and NF2 in mouse BMDMs and RAW264.7 macrophages following treatment with vehicle (Control), LPS, or LPS + rFGF15 (**B**) or LPS + rFGF15, LPS + rFGF15 + si-NC, or LPS + rFGF15 + si-FGFR4 (**C**) by western blot analysis. **D**, **E** Detection of MST1/2, p-MOB1, MOB1, p-YAP, and YAP in mouse RAW264.7 macrophages (**D**) and BMDMs (**E**) following treatment with vehicle (Control), LPS, LPS + rFGF15, LPS + rFGF15 + si-NC, or LPS + rFGF15 + si-FGFR4 by western blot analysis. **F** Detection of the interaction between FGFR4 and NF2 in mouse RAW264.7 macrophages overexpressing the wildtype NF2 (NF2-WT) or phospho-null NF2 mutant (NF2-Mut) following treatment with LPS + rFGF15 by Co-IP. **G** Detection of MST1/2, p-MOB1, MOB1, p-YAP, and YAP in mouse RAW264.7 macrophages overexpressing NF2-WT or NF2-Mut following treatment with LPS + rFGF15 by western blot analysis. *n* = 6, *p < 0.05.

FGF15 has been shown to stimulate hepatic FGFR4 to recruit and phosphorylate NF2, which in turn activates the Hippo kinases MST1/2, suppressing liver tumorigenesis [14]. MOB1 and YAP are key components of the Hippo signaling cascade. The Hippo kinases MST1/2 phosphorylate and activate MOB1, which in turn phosphorylates YAP. This leads to YAP’s retention in the cytoplasm, preventing its nuclear translocation and the expression of its target genes. Given the close integration of the NF2-Hippo pathway with glucose metabolism [13], we speculated that rFGF15 inhibited glycolysis in septic macrophages by modulating the NF2-Hippo pathway. Indeed, the Co-IP results revealed direct association between FGFR4 and NF2 in mouse BMDMs and RAW264.7 macrophages (Fig. 4A). LPS stimulation resulted in reduced levels of phospho-NF2 (p-NF2), MST1/2, phospho-MOB1 (p-MOB1), and phospho-YAP (p-YAP) (Fig. 4B–E), indicating inhibition of the NF2-Hippo pathway. Treatment with rFGF15 restored levels of these proteins, an effect abolished by FGFR4 knockdown (Fig. 4B–E). We induced in vitro BMDM polarization toward M2 using IL-4/IL-13, demonstrating that IL-4/IL-13 stimulated NF2 and MOB1 phosphorylation without affecting MST1, MST2, or YAP phosphorylation. In contrast, rFGF15 treatment increased NF2/MOB1 phosphorylation and MST1/MST2 expression while maintaining unchanged YAP phosphorylation (Fig. S3A, S3B). This indicated the activation of NF2-Hippo signaling in macrophages by FGF15. Further Co-IP experiments with the phospho-null (Y207F) NF2 mutant verified that NF2 phosphorylation was required for FGFR4-NF2 association and the activation of the Hippo pathway (Fig. 4F, G). Collectively, these results demonstrated that FGF15 activates FGFR4 in septic macrophages, leading to the recruitment and phosphorylation of NF2, and subsequent activation of the Hippo pathway.

### FGF15/FGFR4 signaling inhibits glycolysis, H3K18 lactylation, M1 polarization, and inflammation of septic macrophages through the NF2-Hippo pathway

**Fig. 5:**
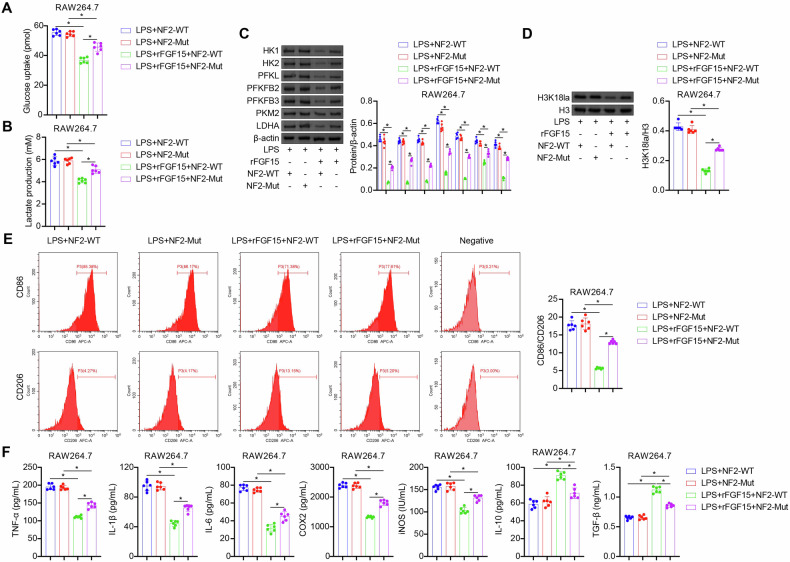
FGF15/FGFR4 signaling inhibits glycolysis, H3K18 lactylation, M1 polarization, and inflammation of septic macrophages through the NF2-Hippo pathway. **A**–**F** Assessment of glucose uptake (**A**), lactate production (**B**), glycolytic enzyme expression (**C**), H3K18 lactylation (**D**), M1/M2 polarization (**E**), and pro- and anti-inflammatory mediator production (**F**) in mouse RAW264.7 macrophages overexpressing the wildtype NF2 (NF2-WT) or phospho-null NF2 mutant (NF2-Mut) following treatment with LPS or LPS + rFGF15. *n* = 6, *p < 0.05.

Having demonstrated that FGF15/FGFR4 signaling activates the NF2-Hippo pathway in septic macrophages, we hypothesized that FGF15 inhibits glycolysis, thereby reducing lactate production and histone lactylation in these cells, through modulation of the NF2-Hippo pathway. To verify this hypothesis, we assessed the effects of phospho-null NF2 mutant by NF2-Mut transfection (Fig. 5A). NF2-Mut abolished the inhibitory effects of rFGF15 on LPS-induced glucose uptake, glycolytic enzyme expression, lactate production, and H3K18 lactylation in mouse RAW264.7 macrophages (Fig. 5A–D). NF2-Mut also eliminated the inhibitory effects of rFGF15 on LPS-induced M1 polarization and cellular inflammation (Fig. 5E, F). Similar to NF2 mutant, NF2 knockdown by si-NF2 transfection abolished the inhibitory effects of rFGF15 on LPS-induced glucose uptake, lactate production, H3K18 lactylation, M1 polarization, and cellular inflammation in mouse BMDMs and RAW264.7 macrophages (Fig. S4A–S4G). These data collectively demonstrated that FGF15/FGFR4 signaling inhibits glycolysis, H3K18 lactylation, M1 polarization, and inflammation in septic macrophages by activating the NF2-Hippo pathway.

### FGF15/FGFR4 signaling suppresses M1 polarization of septic macrophages through NF2-dependent downregulation of H3K18la-driven *Irf7* expression

**Fig. 6:**
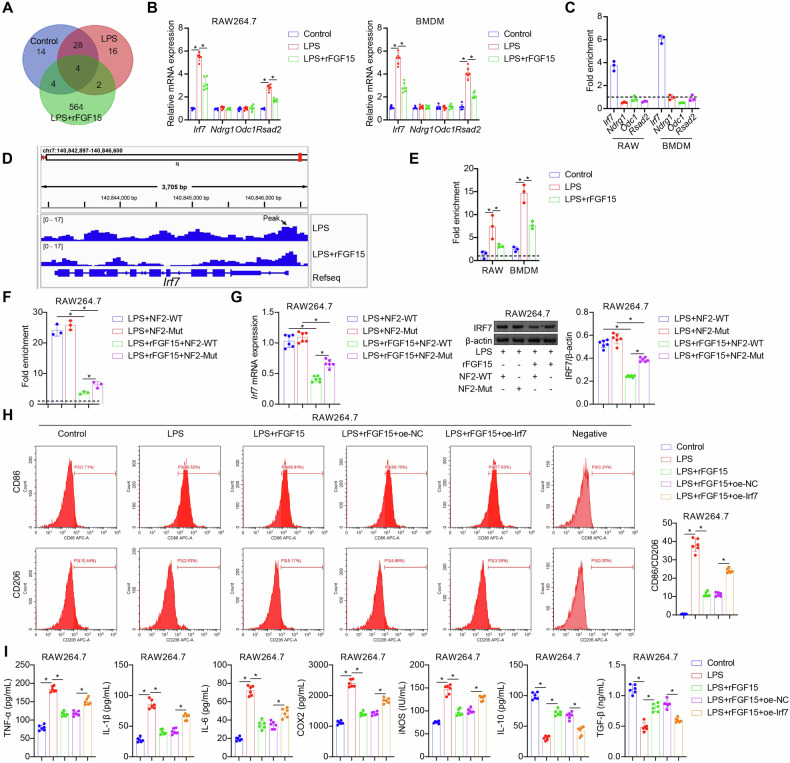
FGF15/FGFR4 suppresses H3K18la-driven *Irf7* expression and inflammatory responses in septic macrophages via NF2. **A** Venn diagram showing the identification of DEGs between mouse BMDMs subjected to specific treatments by RNA-seq. **B** Detection of *Irf7, Ndrg1*, *Odc1*, and *Rsad2* mRNA expression in mouse BMDMs and RAW264.7 macrophages following treatment with vehicle (Control), LPS, or LPS + rFGF15 by qRT-PCR. **C** Fold changes in H3K18la enrichment at the promoter regions of *Irf7, Ndrg1*, *Odc1*, and *Rsad2* in mouse BMDMs and RAW264.7 macrophage in response to LPS stimulation by ChIP-qPCR analysis. **D** Dectection of H3K18la enrichment at the promoter regions of *Irf7* in mouse BMDMs following treatment with LPS or LPS + rFGF15 using the CUT&Tag assay. **E** Fold changes in H3K18la enrichment at the promoter region of *Irf7* in mouse BMDMs and RAW264.7 macrophages following treatment with vehicle (Control), LPS, or LPS + rFGF15 by ChIP-qPCR analysis. **F** Fold changes in H3K18la enrichment at the promoter region of *Irf7* in RAW264.7 macrophages overexpressing the wildtype NF2 (NF2-WT) or phospho-null NF2 mutant (NF2-Mut) following treatment with LPS or LPS + rFGF15 by ChIP-qPCR analysis. **G** Detection of Irf7 mRNA and protein expression in RAW264.7 macrophages overexpressing the wildtype NF2 (NF2-WT) or phospho-null NF2 mutant (NF2-Mut) following treatment with LPS or LPS + rFGF15 by qRT-PCR and western blot analysis. **H**, **I** Assessment of M1/M2 polarization (**H**) and pro- and anti-inflammatory mediator production (**I**) in wildtype and Irf7-overexpressing mouse RAW264.7 macrophages following treatment with LPS or LPS + rFGF15 by flow cytometry and ELISA. *n* = 6, *p < 0.05.

Histone lactylation is an epigenetic modification that regulates gene expression in macrophages [8, 9]. To identify genes influenced by histone lactylation in septic macrophages, we assessed gene expression in control, LPS-stimulated, and rFGF15-treated, LPS-stimulated mouse BMDMs by RNA-seq. A total of four genes were identified as differentially expressed genes (DEGs) among the following comparisons: control vs. LPS, LPS vs. LPS + rFGF15, and control vs. LPS + rFGF15 (Fig. 6A). Out of the four DEGs identified by RNA-seq, Irf7 and radical S-adenosyl methionine domain containing 2 (Rsad2), both of which play key roles in type I interferon response [22], were verified by qRT-PCR. LPS induced both *Irf7* and *Rsad2* mRNA expression in mouse BMDMs and RAW264.7 macrophages, effects inhibited by rFGF15 treatment (Fig. 6B). Subsequent ChIP-qPCR analysis demonstrated increased enrichment of H3K18la at the promoter region of *Irf7*, but not Rsad2, in response to LPS stimulation (Fig. 6C). To identify H3K18la-regulated downstream genes in BMDMs, we performed CUT&Tag assays on LPS-stimulated BMDMs treated with or without rFGF15. We observed H3K18la enrichment at the *Irf7* gene promoter after LPS stimulation, which was attenuated by rFGF15 treatment (Fig. 6D). This was validated through ChIP-qPCR analysis in both mouse BMDMs and RAW264.7 macrophages (Fig. 6E). Notably, the inhibitory effects of rFGF15 on LPS-induced H3K18la enrichment and *Irf7* expression were attenuated by NF2 knockdown or ectopic expression of phosphor-null NF2 mutant (Figs. 6F, G, S5A–S5C). In addition, Irf7 overexpression in mouse BMDMs and RAW264.7 macrophages partially reversed the inhibitory effects of rFGF15 on LPS-induced M1 polarization and cellular inflammation (Figs. 6H, I, S6A, S6B). These data collectively indicated that FGF15/FGFR4 signaling suppresses M1 polarization of septic macrophages and their inflammatory responses through NF2-dependent inhibition of H3K18la-driven *Irf7* expression.

### FGF15 mitigates multi-organ inflammation and improves survival of septic mice by suppressing Irf7-mediated BMDM activation

**Fig. 7:**
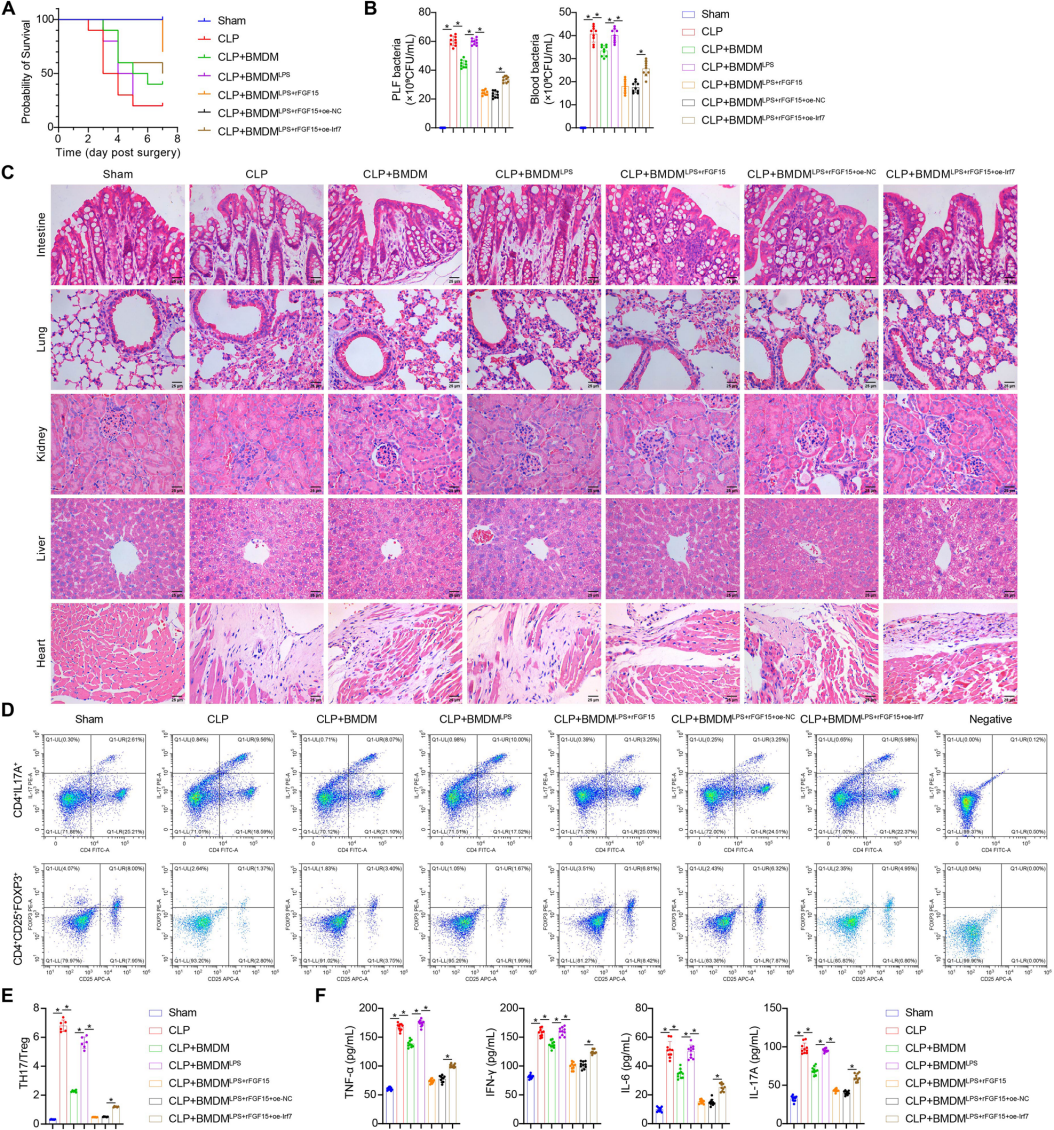
FGF15 protects mice against sepsis-induced multi-organ inflammation by suppressing Irf7-mediated BMDM activation. Macrophage-depleted mice were subjected to the following treatments: Sham, CLP, CLP + transplantation with BMDMs (CLP + BMDM), CLP + transplantation with LPS-stimulated BMDMs (CLP + BMDM^LPS^), CLP + transplantation with rFGF15-treated, LPS-stimulated BMDMs (CLP + BMDM^LPS + rFGF15^), CLP + transplantation with rFGF15-treated, LPS-stimulated BMDMs transfected with empty vector (CLP + BMDM^LPS + rFGF15 + oe-NC^), or CLP + transplantation with rFGF15-treated, LPS-stimulated BMDMs transfected with Irf7-expressing vector (CLP + BMDM^LPS + rFGF15 + oe-Irf7^). **A** Survival analysis. *n* = 10, *p < 0.05. **B** Bacterial loads in peripheral blood and peritoneal lavage fluid (PLF). *n* = 9, *p < 0.05. **C** HE-stained images of the heart, liver, intestine, lung, and kidney showing immune cell infiltration. **D**, **E** Detection of Th17 (CD4 + IL17A + ) and Treg cells (CD4 + CD25 + FOXP3 + ) in peripheral blood by flow cytometry. **D** Density plots. **E** Quantified Th17/Treg ratio. *n* = 6, *p < 0.05. **F** Detection of TNF-α, IFN-γ, IL-6, and IL-17A in peripheral blood by ELISA. *n* = 10, *p < 0.05.

To validate whether our in vitro findings in LPS-stimulated macrophages translate to septic mice, we induced macrophage depletion in vivo using clodronate liposomes, followed by the transplantation of mouse BMDMs subjected to the following treatments: vehicle, LPS, LPS + rFGF15, LPS + rFGF15 + oe-NC, or LPS + rFGF15 + oe-Irf7. Intravenous injection of clodronate liposomes reduced the proportion of bone marrow macrophages (CD11b + F4/80 + ) to approximately 2%, both pre- and post-CLP (Fig. S7). Macrophage-depleted mice showed reduced survival after CLP, which was restored by BMDM transplantation following the order of LPS + rFGF15 ≈ LPS + rFGF15 + oe-NC > LPS + rFGF15 + oe-Irf7 > vehicle > LPS ≈ no transplantation (Fig. 7A). Transplantation of untreated BMDMs effectively reduced bacterial loads in both blood and PLF of macrophage-depleted CLP mice. However, transplantation of LPS-stimulated BMDMs restored bacterial infection, an effect which was alleviated by rFGF15 treatment but worsened by Irf7 overexpression (Fig. 7B). Multi-organ dysfunction represents the most severe complication of sepsis. Immune cell infiltration was observed in the heart, liver, intestine, lung, and kidney in macrophage-depleted CLP mice, which was reduced by BMDM transplantation, correlating with survival (Fig. 7C). Treatment with rFGF15 reduced LPS-induced immune cell infiltration, an effect was reversed by Irf7 overexpression (Fig. 7C). Pulmonary assessment was performed by measuring dry/wet weight ratios. Macrophage-depleted CLP mice exhibited increased lung dry/wet ratios, which decreased following BMDM transplantation. LPS pretreatment exacerbated pulmonary edema, while rFGF15 effectively reduced it; however, this effect was reversed upon Irf7 overexpression (Fig. S8A). For renal function, Scr and BUN levels - indicators of glomerular filtration capacity - were elevated in macrophage-depleted CLP mice. In contrast, BMDM transplantation reduced Scr and BUN levels, which increased again after LPS treatment. Treatment with rFGF15 attenuated LPS-induced Scr and BUN elevations, an effect abolished by Irf7 overexpression (Fig. S8B, S8C). Hepatic evaluation included PT and plasma biomarkers, including ALB, ALT, and TB. BMDM transplantation improved hepatic function by prolonging PT, elevating ALB and TB levels, and reducing ALT, effects reversed by LPS pretreatment. Under LPS conditions, Irf7 overexpression disrupted rFGF15-mediated hepatoprotection (Fig. S8D–S8G). Intestinal barrier integrity was assessed through tight junction proteins (ZO-1, Occludin, Claudin-1). CLP downregulated colonic expression of these proteins in macrophage-depleted mice, which was restored by BMDM transplantation. rFGF15 upregulated LPS-suppressed tight junction proteins, whereas Irf7 overexpression counteracted this effect (Fig. S8H–S8K). Quantitative analysis of serum cardiac biomarkers demonstrated that BMDM transplantation reduced accumulations of BNP, CK-MB, and cTnI in macrophage-depleted CLP mice. Conversely, LPS-upregulated BNP, CK-MB, and cTnI levels were reduced by rFGF15 administration, effects that were reversed with Irf7 overexpression (Fig. S8L–S8N). These in vivo data demonstrated that FGF15 alleviates multi-organ dysfunction and improves survival of septic mice by suppressing Irf7-driven BMDM activation.

M1 polarization of macrophages can affect the balance between proinflammatory Th17 cells and immunosuppressive Treg cells [23]. Compared with macrophage-depleted sham mice, macrophage-depleted CLP mice exhibited a higher ratio of Th17 cells (CD4 + IL-17A + ) to Treg cells (CD4 + CD25 + FOXP3 + ) in peripheral blood, accompanied by increased levels of pro-inflammatory cytokines TNF-α, IFN-γ, IL-6, and IL-17A (Fig. 7D–F). BMDM transplantation reduced the Th17/Treg ratio and pro-inflammatory cytokine levels in macrophage-depleted CLP mice, mirroring the observed patterns of immune cell infiltration (Fig. 7D–F). Collectively, these data indicated that FGF15 mitigates multi-organ inflammation of septic mice by suppressing Irf7-driven BMDM activation.

## Discussion

If left untreated, sepsis can progress rapidly to septic shock, causing multiple organ failure and potentially death. A predominant M1 macrophage response plays a key role in driving early septic inflammation and disease progression [4]. In this study, we found that rFGF15 mitigated inflammatory damage and dysfunction in major organs and improved survival of CLP-induced septic mice. Mechanistic investigations in vitro and in vivo demonstrated that, through its receptor FGFR4, rFGF15 inhibits H3K18la-driven *Irf7* expression through NF2-Hippo to suppress septic macrophage M1 polarization and inflammation. These findings highlight the pathogenic role of M1 macrophage polarization in sepsis and support rFGF15 as a potential innovative therapy for this life-threatening condition.

Previous studies have found that FGFR4 deficiency or inactivation in mouse BMDMs aggravated LPS-induced M1 polarization and inflammation, and myeloid-specific FGFR4 deletion exacerbated colitis in mice [15]. RNA-seq in combination with KEGG pathway enrichment analysis revealed that FGFR4-deficiency in mouse BMDMs led to pentraxins3-mediated activation of the complement cascade through NF-κB [15]. In our in vitro experiments, we found that rFGF15 stimulated FGFR4 to bind and phosphorylate NF2 in LPS-stimulated mouse BMDMs and RAW264.7 macrophages, thereby activating the Hippo pathway and subsequently inhibiting glycolysis, lactate production, and the lactylation of histone H3K18. This led to reduced *Irf7* expression, resulting in decreased M1 polarization and associated inflammation. The interactions between FGFR4 and p-NF2 and between H3K18la and the *Irf7* promoter were confirmed by Co-IP and ChIP-qPCR analysis, respectively. Importantly, our in vivo BMDM transplantation experiments demonstrated that rFGF15-mediated inhibition of BMDM M1 polarization provided complete protection against CLP-induced sepsis in mice. These findings suggest that rFGF15 may serve as an effective early intervention for sepsis by modulating macrophage polarization.

The Hippo pathway is an evolutionarily conserved signaling network that regulates numerous biological processes. As such, its dysregulation contributes to the pathogenesis of a variety of diseases, such as cancer and immune dysfunctions [24, 25]. Specifically, MST1/2-deficient mice exhibited increased susceptibility to CLP-induced sepsis [26]. Mechanistically, MST1 and MST2 promoted ROS production and phagocytic activity of mouse BMDMs by regulating mitochondrion-phagosome juxtaposition [26]. Moreover, YAP1 knockout aggravated CLP-induced liver and lung inflammatory injury by accelerating ferroptosis [27, 28]. In this study, we discovered that rFGF15-induced activation of the NF2-Hippo pathway in septic macrophages downregulated glycolysis and lactate production, leading to a reduction in H3K18 lactylation and H3K18la-driven *Irf7* expression, ultimately attenuating M1 polarization. Our findings further underscore the pivotal role of the Hippo pathway in sepsis, specifically in controlling gene expression by modulating glycolysis and histone lactylation in septic macrophages.

In the early stage of sepsis, pro-inflammatory stimuli promote macrophage polarization toward the M1-like phenotype, which predominantly depends on aerobic glycolysis to sustain its energy demands for phagocytosis and inflammatory responses [6, 7]. Suppression of glycolysis through inhibition of sphingosine kinase 1 promoted M2 macrophage polarization and reduced septic inflammation in vitro and in vivo [29]. Histone lactylation, an epigenic modefication directly linked to glycolysis, has been increasingly recognized for its role in regulating macrophage polarization, particularly in tumorigenesis [9]. However, how histone lactylation affects macrophage polarization in sepsis remains largely unclear. In this study, we discovered that septic stimuli enhanced glycolysis and histone lactylation in mouse macrophages in vitro and in vivo, resulting in increased H3K18la enrichment at the promoter region of *Irf7*, a transcription factor that plays a crucial role in M1 polarization of septic macrophages [30]. FGF15 protected against septic inflammation by inhibiting the signaling cascade of glycolysis, histone lactylation, Irf7 induction, and M1 polarization through NF2-Hippo activation. In line with previous reports [31, 32], our findings further supported targeting macrophage polarization as a promising therapeutic approach for mitigating sepsis.

This study had several limitations. In addition to histones, lactylation modifications are also widely present in various non-histones to coordinate transcription [33]. To our knowledge, only a few non-histone proteins - HMGB1 [34], Fis1 [35], and Ezrin [36] - have been reported to undergo lactylation in the context of sepsis. We did not explore the differential roles of histone versus non-histone lactylation in sepsis and the role of non-histone proteins in macrophages. Secondly, although we verified that H3K18la binds to the promoter region of *Irf7* in septic macrophages, we did not identify the specific binding site sequences. Thirdly, our mechanistic studies investigated only the regulation of glycolysis and histone lactylation mediated by the NF2-Hippo pathway. In future studies, we plan to identify putative specific H3K18la binding sites within the *Irf7* promoter region through bioinformatics analysis. We also would investigate how mutations at the putative H3K18la-binding sites would affect *Irf7* expression and septic inflammation. Previous studies have shown that FGFR4 deficiency in mouse BMDMs aggravated LPS-induced M1 polarization and inflammation by activation of the complement cascade through NF-κB [15]. The functional diversity of Hippo signaling in macrophage activities suggests that it may be involved in different cascades [37, 38]. Thus, we plan to investigate the effects of rFGF15-Hippo on the complement system in septic macrophages in future experiments.

In conclusion, rFGF15 mitigated multi-organ inflammation and improved survival of CLP-induced septic mice by suppressing macrophage M1 polarization. Mechanistically, rFGF15 activated the NF2-Hippo pathway through FGFR4 in septic microphages, leading to reduced glycolysis, lactate production, H3K18 lactylation, and H3K18la-driven *Irf7* expression. FGF15 holds promise as a potential innovative therapy for sepsis.

## Supplementary information

Original Western Blot Images in Figure 1-2

Original Western Blot Images in Figure 4-6

Original Western Blot Images in Supplementary Figures

Supplementary Materials

## Data Availability

Data will be made available on request. The raw data can be found in NCBI (https://www.ncbi.nlm.nih.gov/bioproject/PRJNA1281990).
