# Supplementary figures and images for: FGF15/FGFR4 signaling suppresses M1 macrophage polarization and multi-organ inflammation in septic mice by inhibiting H3K18 lactylation-driven Irf7 expression through NF2-Hippo activation
Source: Cell Death Dis. 2025 Aug 19;16(1):628. doi: 10.1038/s41419-025-07962-w (PMC12361455; doi:10.1038/s41419-025-07962-w)

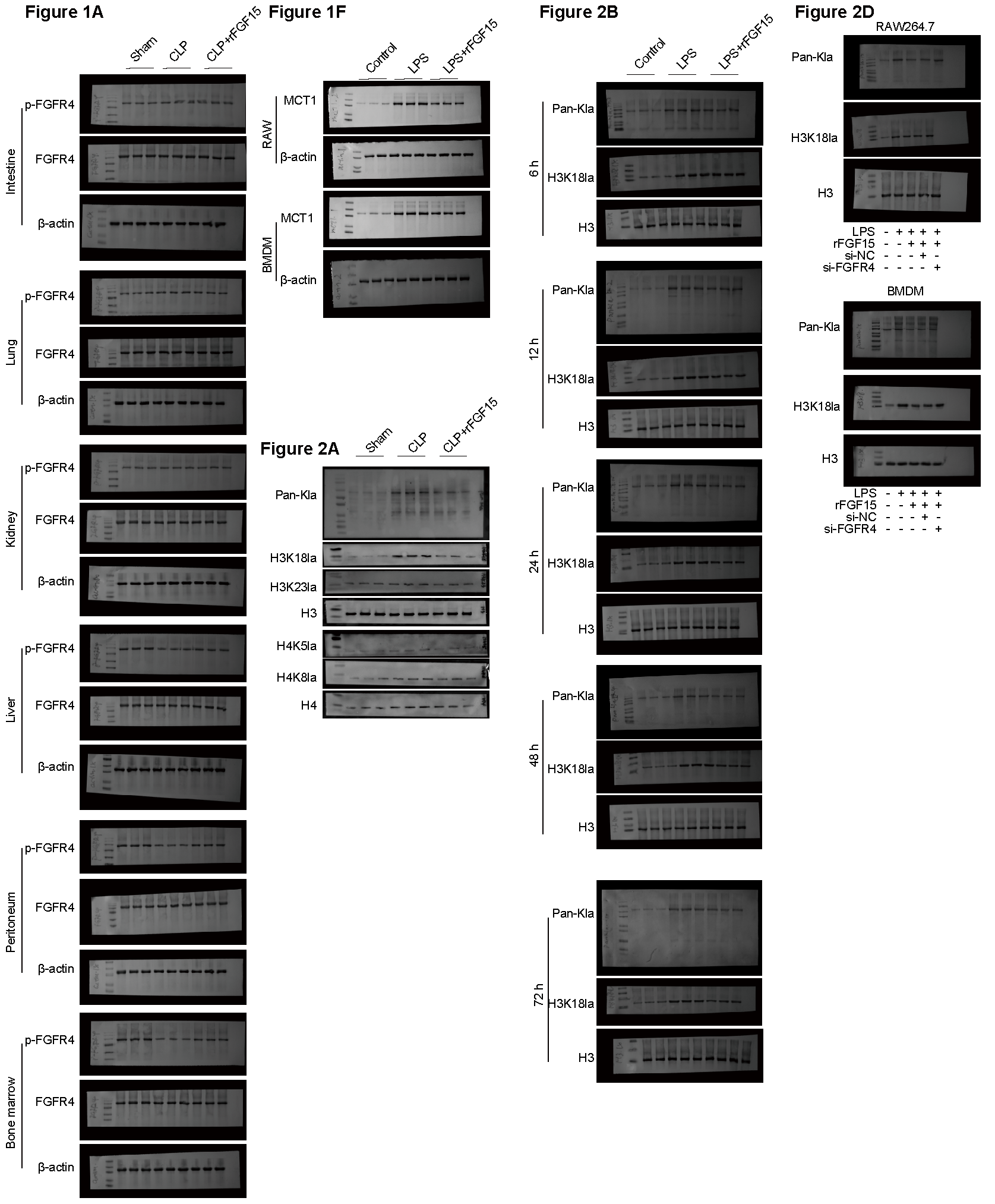

Supplement: Supplementary file 1 — Original Western Blot Images in Figure 1-2 [file 41419_2025_7962_MOESM1_ESM.tif]

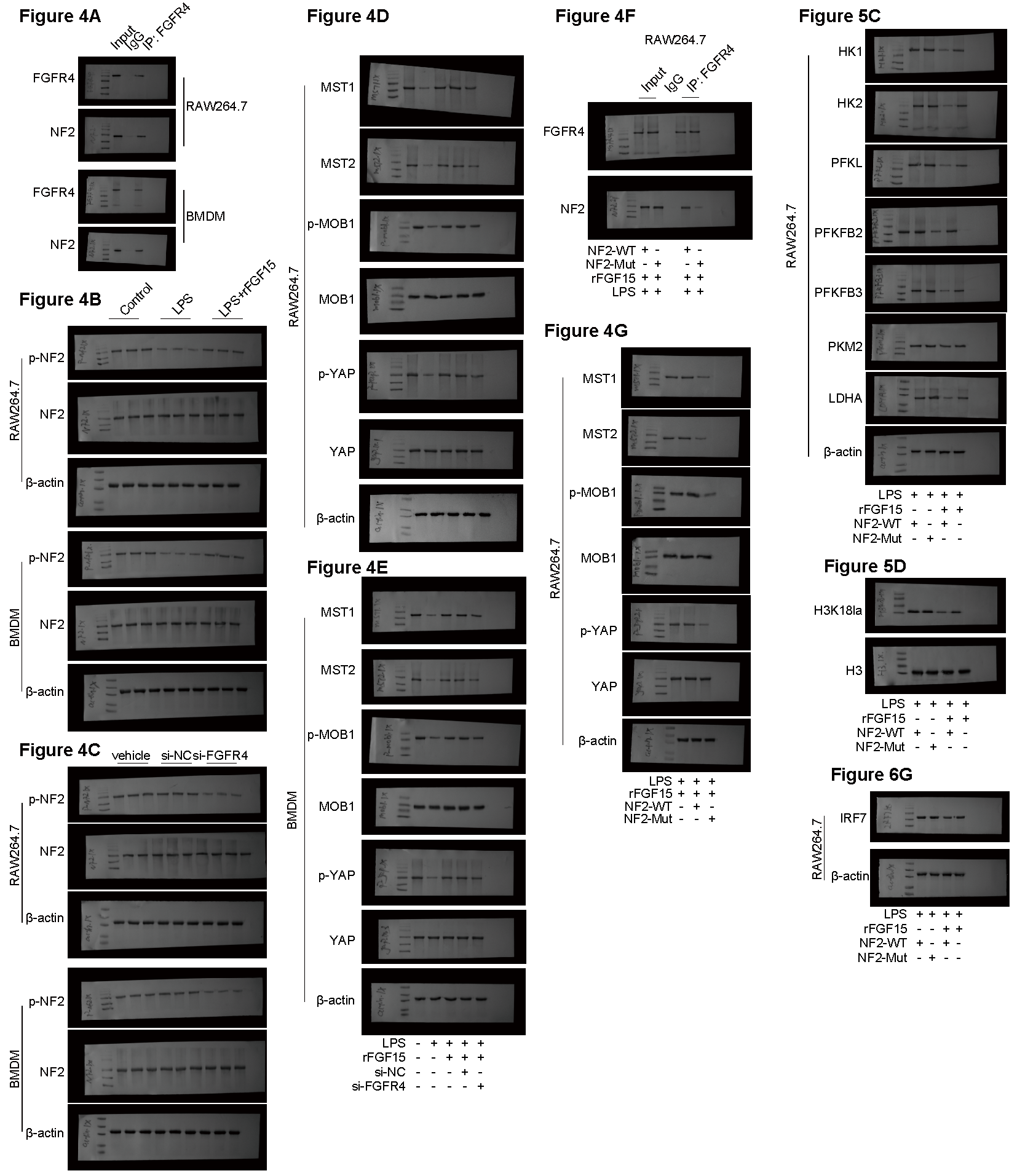

Supplement: Supplementary file 2 — Original Western Blot Images in Figure 4-6 [file 41419_2025_7962_MOESM2_ESM.tif]

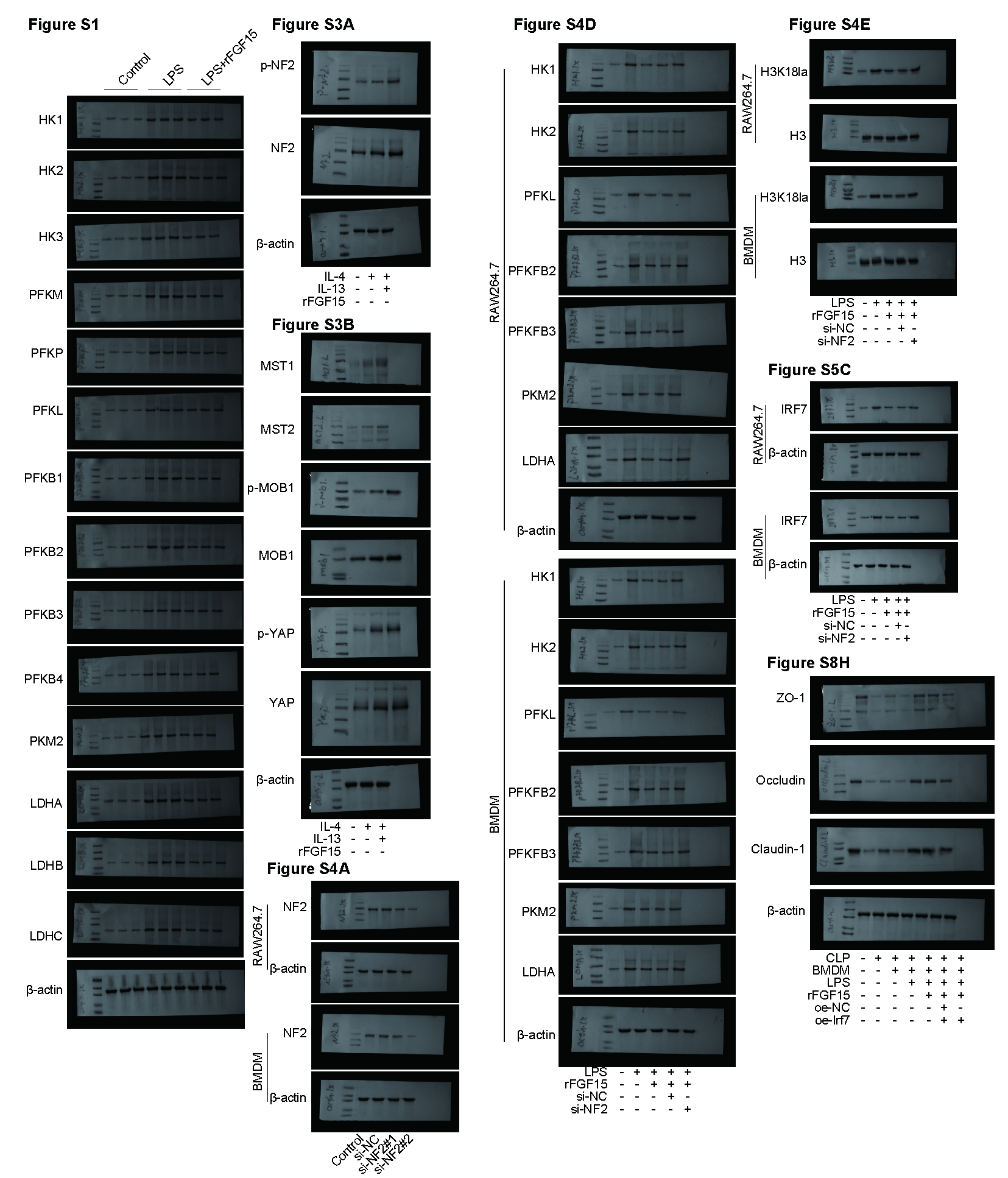

Supplement: Supplementary file 3 — Original Western Blot Images in Supplementary Figures [file 41419_2025_7962_MOESM3_ESM.tif]
