# Supplementary material for: FGF15/FGFR4 signaling suppresses M1 macrophage polarization and multi-organ inflammation in septic mice by inhibiting H3K18 lactylation-driven Irf7 expression through NF2-Hippo activation
Source: Cell Death Dis. 2025 Aug 19;16(1):628. doi: 10.1038/s41419-025-07962-w (PMC12361455; doi:10.1038/s41419-025-07962-w)

Supplementary Materials

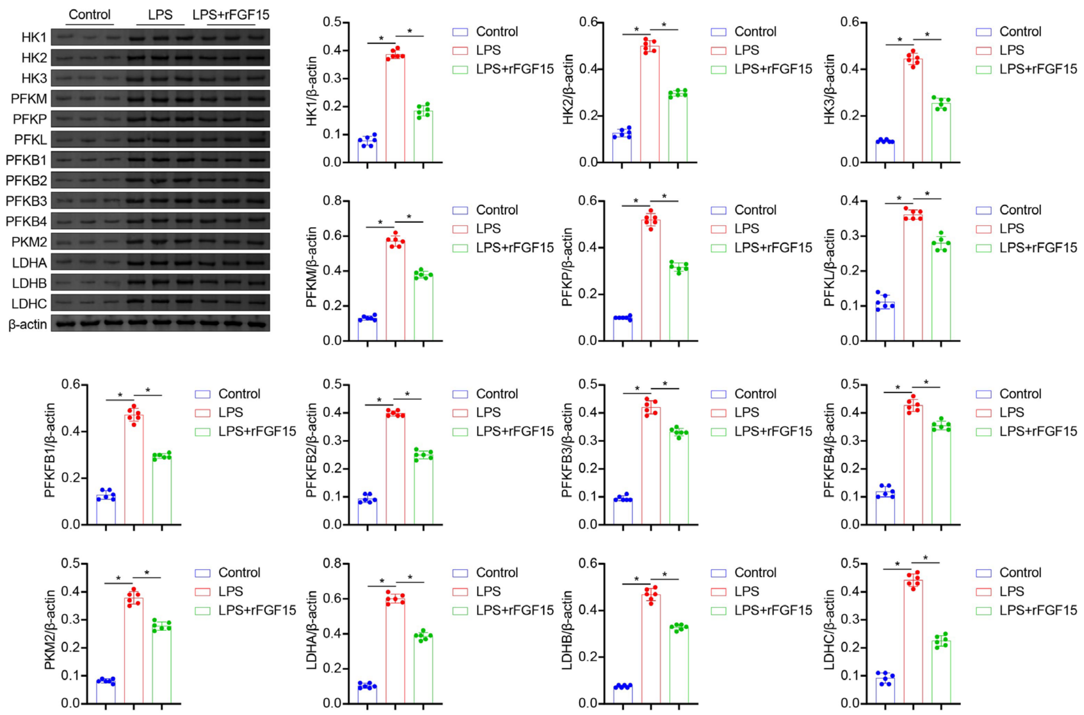

Fig. S1. Assessment of glycolytic enzyme expression in control, LPS-stimulated (LPS), and rFGF15-treated, LPS-stimulated (LPS + rFGF15) mouse BMDMs.

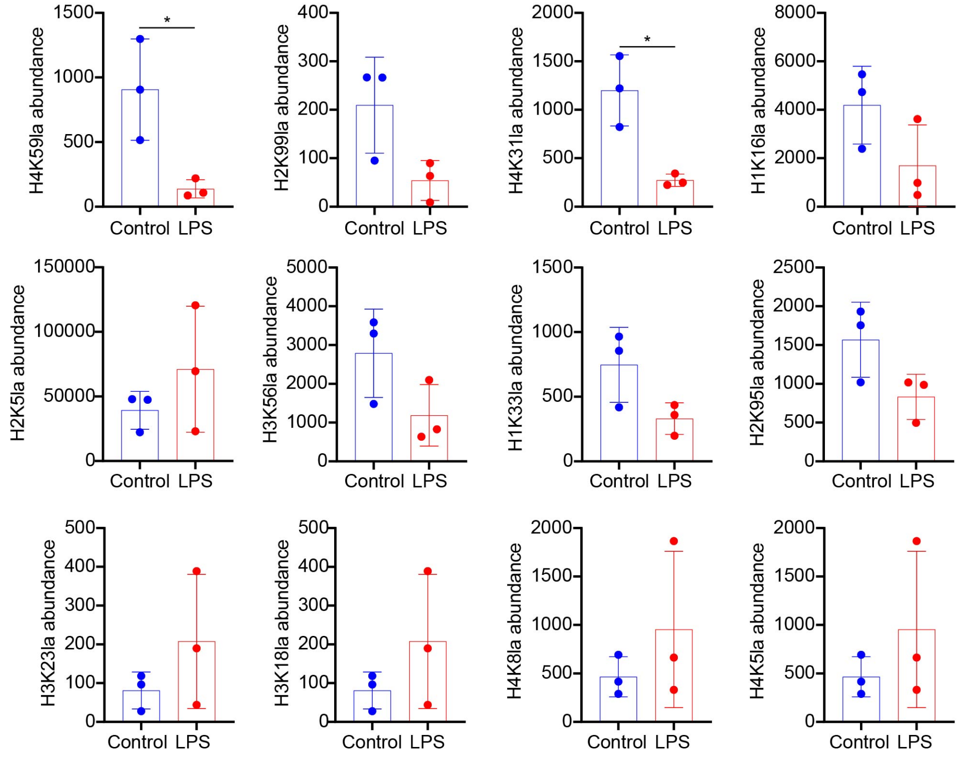

Fig. S2. LC–MS/MS analysis of levels of lactylated histones in control and LPS-stimulated mouse BMDMs.

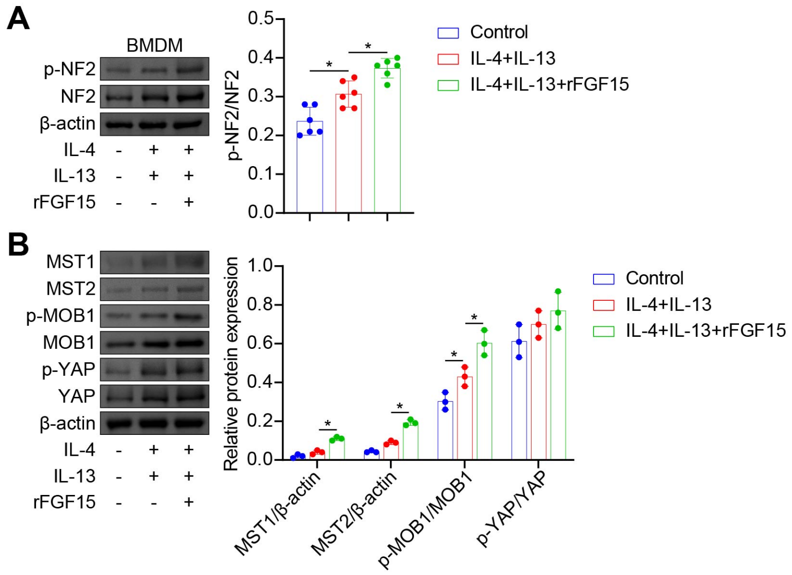

Fig. S3. rFGF15 augments IL-4/IL-13-induced activation of the NF2-Hippo pathway in mouse BMDMs. (A) Detection of p-NF2 and NF2 in mouse BMDMs following

treatment with vehicle (Control), IL4 + IL-13, or IL4 + IL-13 + rFGF15 by western blot analysis. (B) Detection of MST1/2, p-MOB1, MOB1, p-YAP, and YAP in mouse BMDMs following treatment with vehicle (Control), IL4 + IL-13, or IL4 + IL-13 + rFGF15 by western blot analysis. n = 3, $*p < 0.05$.

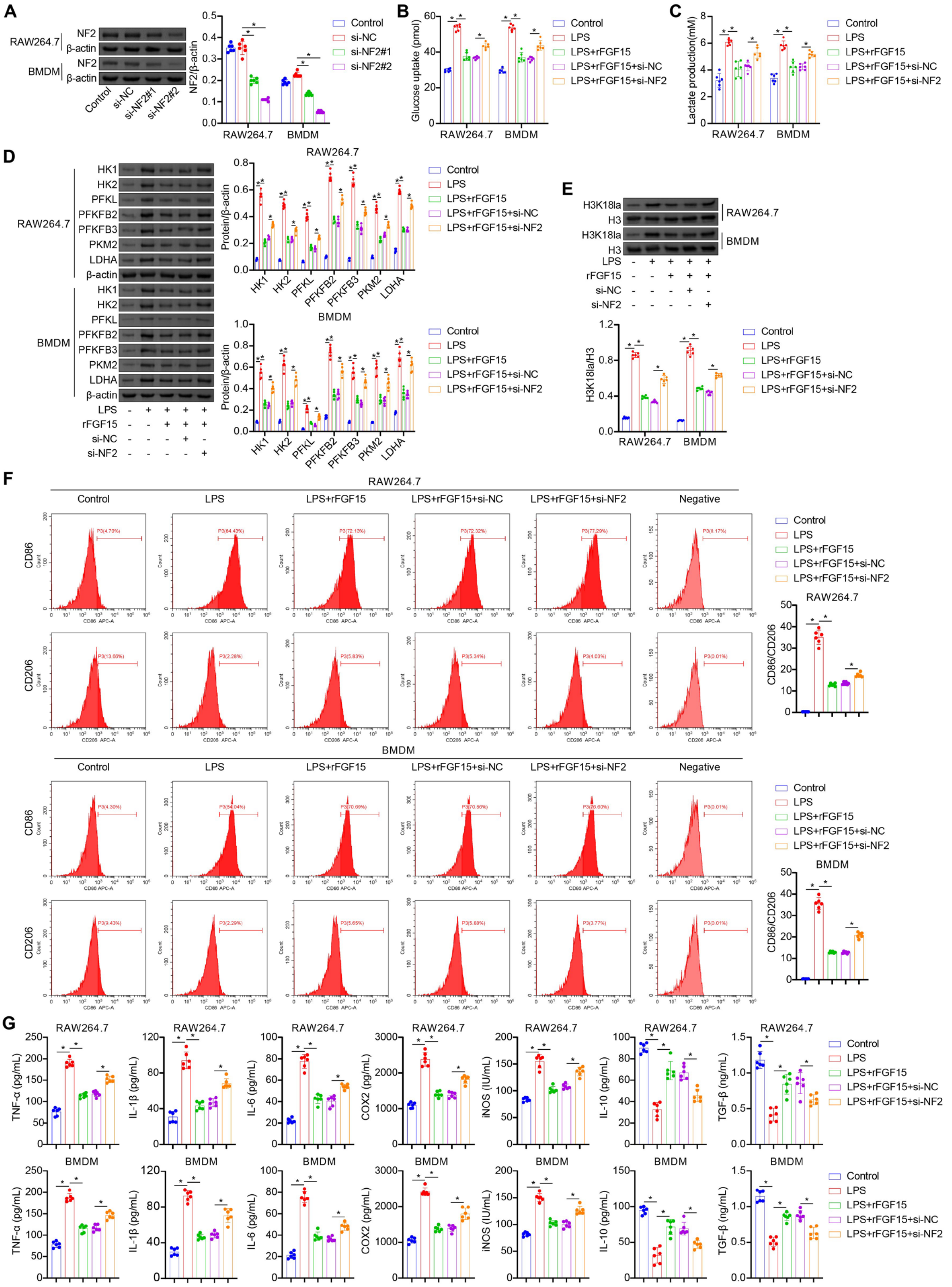

Fig. S4. NF2 knockdown reverses the effects of FGF15/FGFR4-mediated glycolysis, H3K18 lactylation, M1 polarization, and inflammation of septic macrophages. (A) Evaluation of NF2-silencing plasmid transfection efficiency in mouse BMDMs and RAW264.7 macrophages. (B–G) Assessment of glucose uptake

(B), lactate production (C), glycolytic enzyme expression (D), H3K18 lactylation (E), M1/M2 polarization (F), and pro- and anti-inflammatory mediator production (G) in mouse BMDMs and RAW264.7 macrophages following treatment with vehicle (Control), LPS, LPS + rFGF15, LPS + rFGF15 + si-NC, or LPS + rFGF15 + si-NF2. n = 6, *$p < 0.05$.

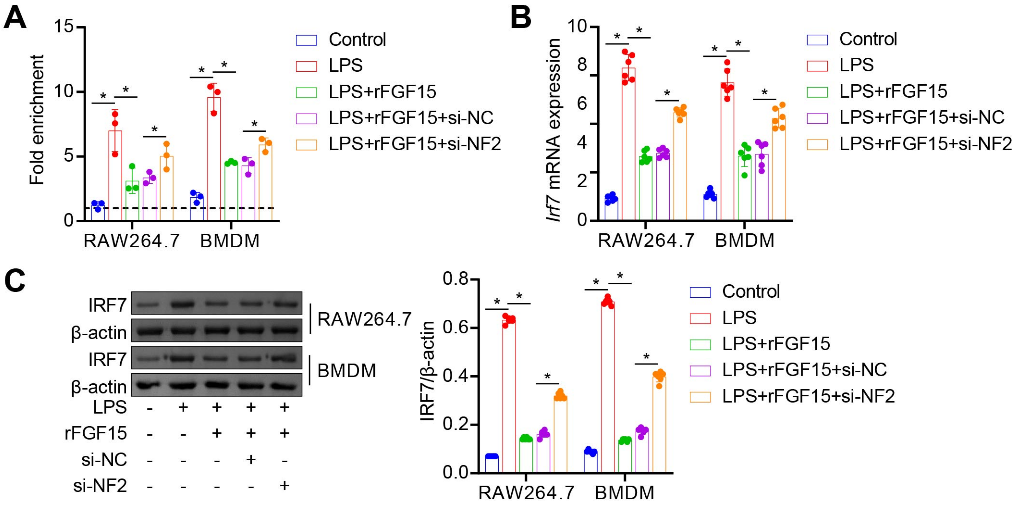

Fig. S5. FGF15/FGFR4 signaling suppresses H3K18la enrichment and Irf7 expression via NF2. (A) Fold changes in H3K18la enrichment at the promoter region of *Irf7* in mouse BMDMs and RAW264.7 macrophages following treatment with vehicle (Control), LPS, LPS + rFGF15, LPS + rFGF15 + si-NC, or LPS + rFGF15 + si-NF2 by ChIP-qPCR analysis. (B, C) Detection of Irf7 mRNA (B) and protein expression (C) in mouse BMDMs and RAW264.7 macrophages following treatment with vehicle (Control), LPS, LPS + rFGF15, LPS + rFGF15 + si-NC, or LPS + rFGF15 + si-NF2 by qRT-PCR and western blot analysis. n = 6, *$p < 0.05$.

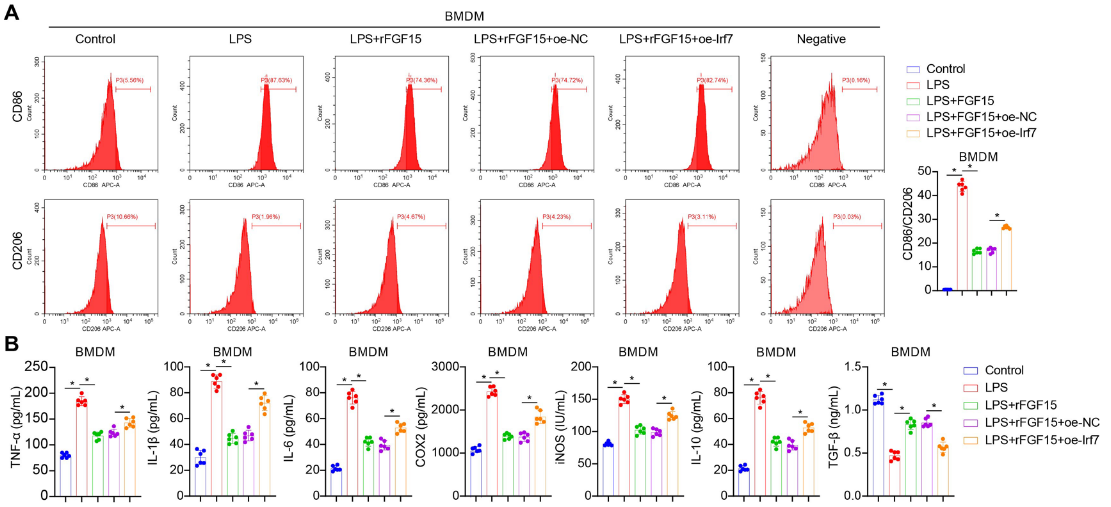

Fig. S6. FGF15/FGFR4 signaling suppresses M1 polarization of septic macrophages and their inflammatory responses through Irf7 inhibition. (A) Assessment of M1/M2 polarization in wildtype and Irf7-overexpressing mouse BMDMs and RAW264.7 macrophages following treatment with LPS or LPS + rFGF15 by flow cytometry. (B) Assessment of pro- and anti-inflammatory mediator production in wildtype and Irf7-overexpressing mouse BMDMs and RAW264.7 macrophages following treatment with LPS or LPS + rFGF15 by ELISA. n = 6, **p* < 0.05.

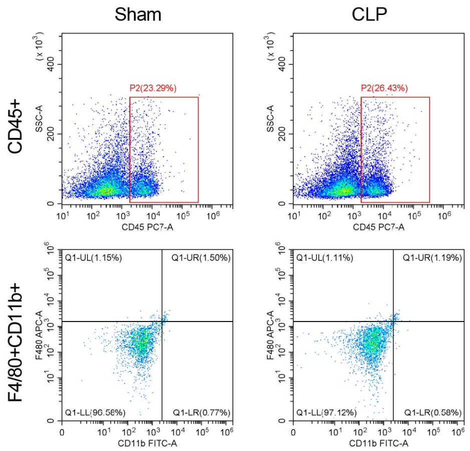

Fig. S7. The proportion of bone marrow macrophages in Sham and CLP mice after intravenous injection of clodronate liposomes.

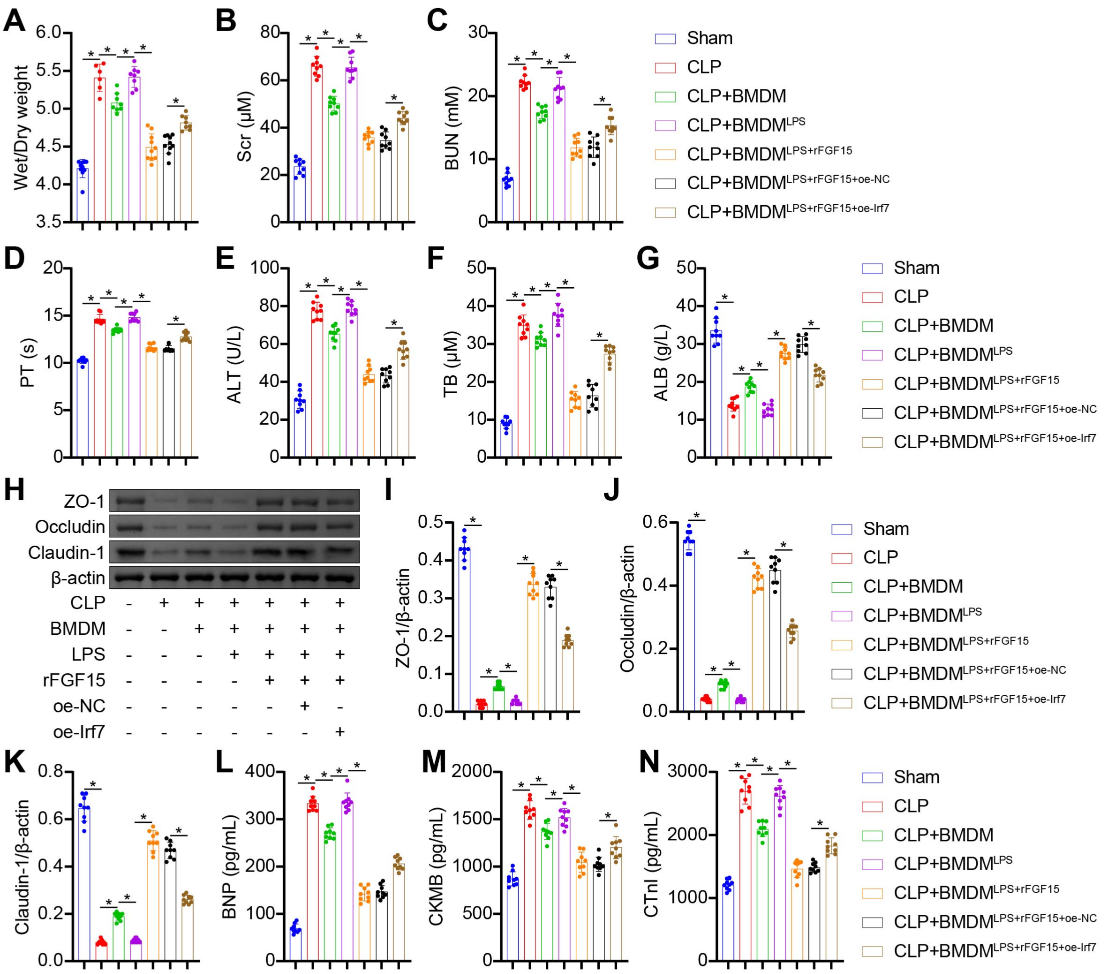

Fig. S8. FGF15 mitigates multi-organ dysfunction in septic mice by suppressing Irf7-driven BMDM activation. Macrophage-depleted mice were subjected to the following treatments: Sham, CLP, CLP + transplantation with BMDMs (CLP + BMDM), CLP + transplantation with LPS-stimulated BMDMs (CLP + BMDM^{LPS}), CLP + transplantation with rFGF15-treated, LPS-stimulated BMDMs (CLP + BMDM$^{LPS + rFGF15}$), CLP + transplantation with rFGF15-treated, LPS-stimulated BMDMs transfected with empty vector (CLP + BMDM$^{LPS + rFGF15 + oe\text{-}NC}$), or CLP + transplantation with rFGF15-treated, LPS-stimulated BMDMs transfected with Irf7-expressing vector (CLP + BMDMLPS + rFGF15 + oe-Irf7). (A) Lung dry/wet weight

ratios for assessing lung edema. (B, C) Detection of serum creatinine (Scr) (B) and blood urea nitrogen (BUN) (C) in peripheral blood for assessing renal function. (D) Detection of prothrombin time (PT). (E–G) Detection of alanine aminotransferase (ALT) (E), total bilirubin (TB) (F), and albumin (ALB) (G) in peripheral blood by ELISA for assessing liver function. (H–K) Detection of tight junction proteins (ZO-1, Occludin, Claudin-1) in colonic tissues by western blot analysis for assessing intestinal barrier integrity. (H) Immunoblot images. (I) Quantified ZO-1. (J) Quantified Occludin. (K) Quantified Claudin-1. (L–N) Detection of B-type natriuretic peptide (BNP) (L), creatine kinase-MB isoenzyme (CK-MB) (M), and cardiac troponin I (cTnI) (N) in peripheral blood by ELISA for assessing cardiac function. n = 9, *$p < 0.05$.

Supplementary Table 1. Primary and secondary antibodies for western blot analysis.

Protein	Fold dilution	Catalog number	Manufacturer
Rabbit anti-p-FGFR4	1: 1000	ab192589	Abcam
Rabbit anti-FGFR4	1: 1000	11098-1-AP	Proteintech
Rabbit anti-MCT1	1: 4000	20139-1-AP	Proteintech
Rabbit anti-Pan-Kla	1: 1000	ab96173	Abcam
Rabbit anti-H3K18	1: 1000	PTM-1427RM	PTM BIO
Rabbit anti-H3K23	1: 2000	PTM-1413RM	PTM BIO
Rabbit anti-H4K5	1: 1000	PTM-1407RM	PTM BIO
Rabbit anti-H4K8	1: 1000	PTM-1415RM	PTM BIO

Rabbit anti-H4K12	1: 10000	ab177793	Abcam
Rabbit anti-H3	1: 1000	ab1791	Abcam
Rabbit anti-H4	1: 1000	PTM-1009	PTM BIO
Rabbit anti-NF2	1: 5000	21686-1-AP	Proteintech
Rabbit anti-p-NF2	1: 1000	ab47378	Abcam
Rabbit anti-MST1	1: 2000	22245-1-AP	Proteintech
Rabbit anti-MST2	1: 2000	12097-1-AP	Proteintech
Rabbit anti-MOB1	1: 2000	12790-1-AP	Proteintech
Rabbit anti-p-MOB1	1: 1000	29027-1-AP	Proteintech
Rabbit anti-YAP	1: 10000	13584-1-AP	Proteintech
Rabbit anti-p-YAP	1: 10000	80694-2-RR	Proteintech
Rabbit anti-HK1	1: 1000	15656-1-AP	Proteintech
Rabbit anti-HK2	1: 10000	22029-1-AP	Proteintech
Mouse anti-HK3	1: 10000	67803-1-Ig	Proteintech
Rabbit anti-PFKM	1: 1000	30326-1-AP	Proteintech
Rabbit anti-PFKP	1: 10000	13389-1-AP	Proteintech
Mouse anti-PFKL	1: 10000	68385-1-Ig	Proteintech
Rabbit anti-PFKB1	1: 1000	21718-1-AP	Proteintech
Rabbit anti-PFKB2	1: 1000	ab234865	Abcam
Rabbit anti-PFKB3	1: 1000	13763-1-AP	Proteintech
Rabbit anti-PFKB4	1: 1000	29902-1-AP	Proteintech
Rabbit anti-PKM2	1: 2000	15822-1-AP	Proteintech

Rabbit anti-LDHA	1: 10000	21799-1-AP	Proteintech
Rabbit anti-LDHB	1: 10000	14824-1-AP	Proteintech
Rabbit anti-LDHC	1: 1000	14546-1-AP	Proteintech
Rabbit anti-IRF7	1: 2000	22392-1-AP	Proteintech
Rabbit anti-ZO-1	1: 1000	ab96587	Abcam
Rabbit anti-Occludin	1: 1000	27260-1-AP	Proteintech
Rabbit anti-Claudin-1	1: 1000	ab15098	Abcam
Mouse β-actin	1: 5000	66009-1-Ig	Proteintech
HRP goat anti-mouse IgG	1: 5000	SA00001-1	Proteintech
HRP goat anti-rabbit IgG	1: 6000	SA00001-2	Proteintech

Supplementary Table 2. Primers for qRT-PCR and ChIP-qPCR analysis.

Gene	Forward sequence (5'-3')	Reverse sequence (5'-3')
Irf7	ACAAGGCATCACAGAGTAGTAGCATC	GCCAGTCTCCAAACAGCACTCG
Ndrg1	ATGGTAGAGGGTCTCGTGCT	CGGGTTCATGTCGTTGAGGA
Odc1	CACACTCAAAACCAGCAGGC	ATCCACTGCCCACATGGAAG
Rasd2	TCACACCCAGCAGCAGTTAG	ATACTCACATGAGGCCTGCG
Irf7-chip	CTTTTGACTTTCATTTCGGCTGG	GCAGCTCACAACTGTAACTCC
Ndrg1-chip	CCCGGTTTCCTTTCTGTTGTT	TCTTTCTTCAGGACAGGGCAT
Odc1-chip	TCTTCCTGACCAAGACCTGC	CTCTTCTCTTGGGCACCCT
Rasd2-chip	CAAGACCCCTTCATCACATGC	GCACATTAAGAACCAACATCAGG
β-actin	ATGGTAGAGGGTCTCGTGCT	CGGGTTCATGTCGTTGAGGA

Supplementary file 1. Peptide mapping of specific histone lactylated modification sites

H4K59la

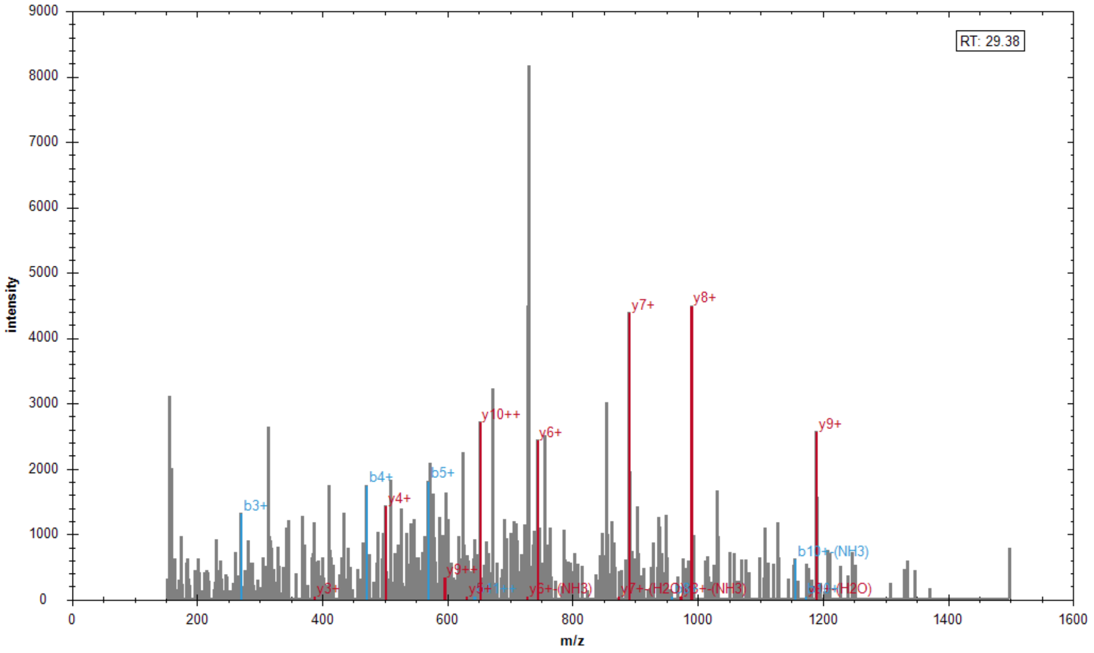

H2K99la

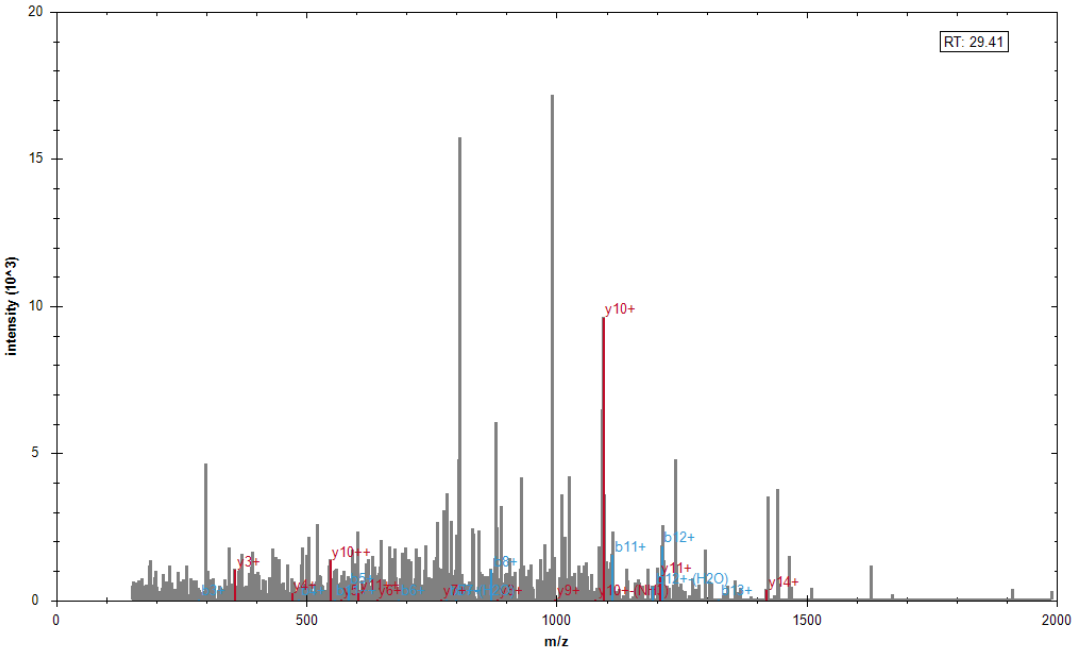

H4K31la

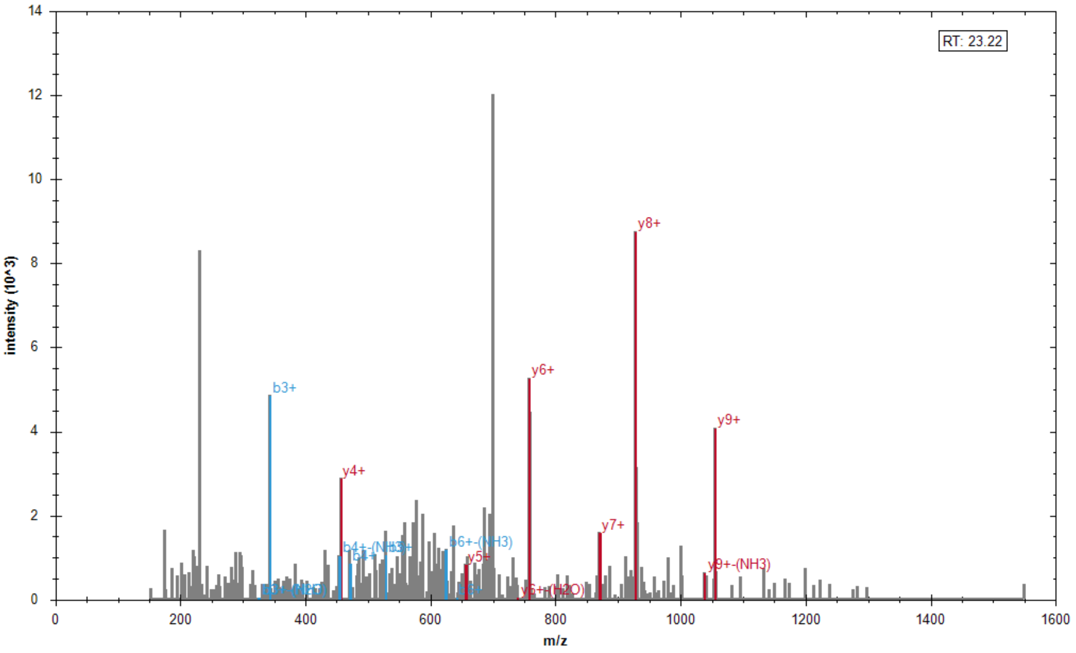

H1K16la

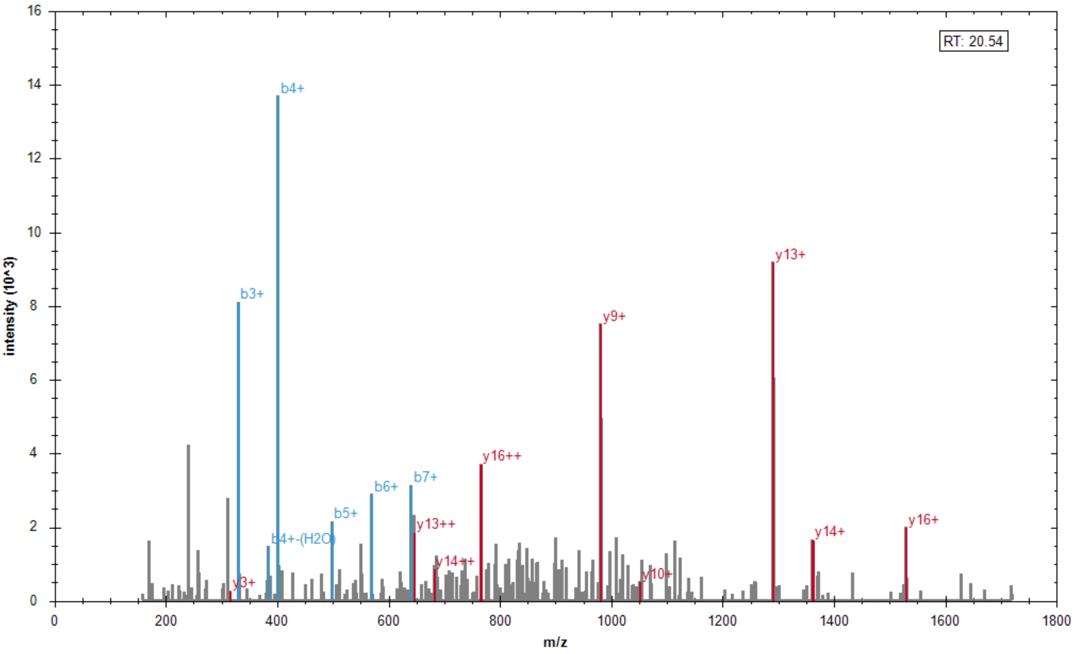

H2K95la

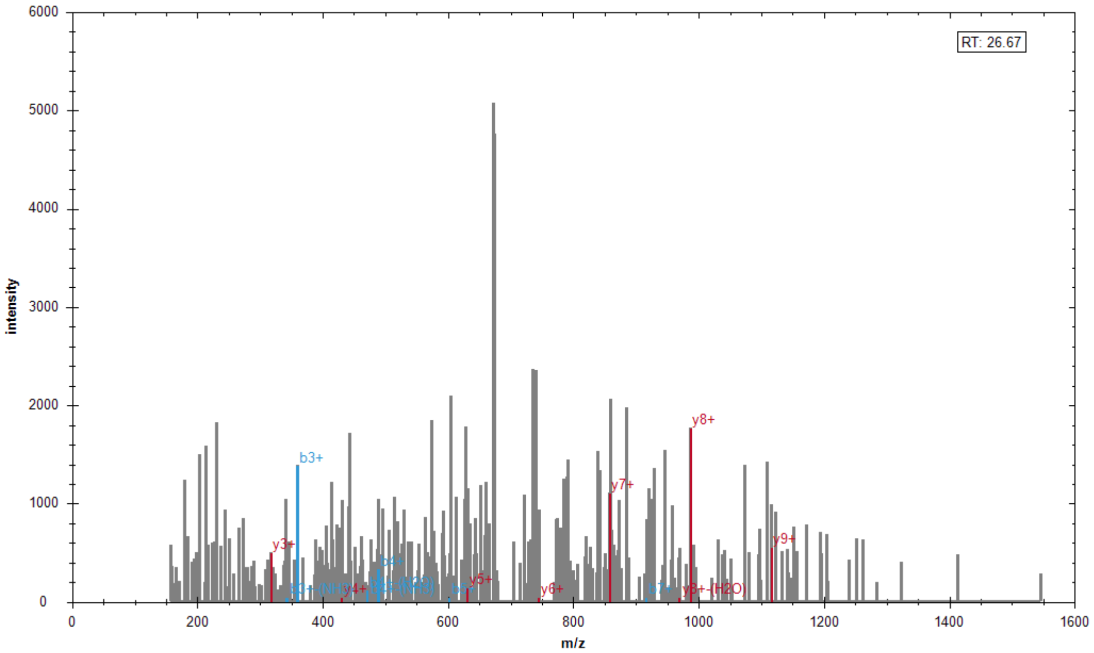

H3K56la

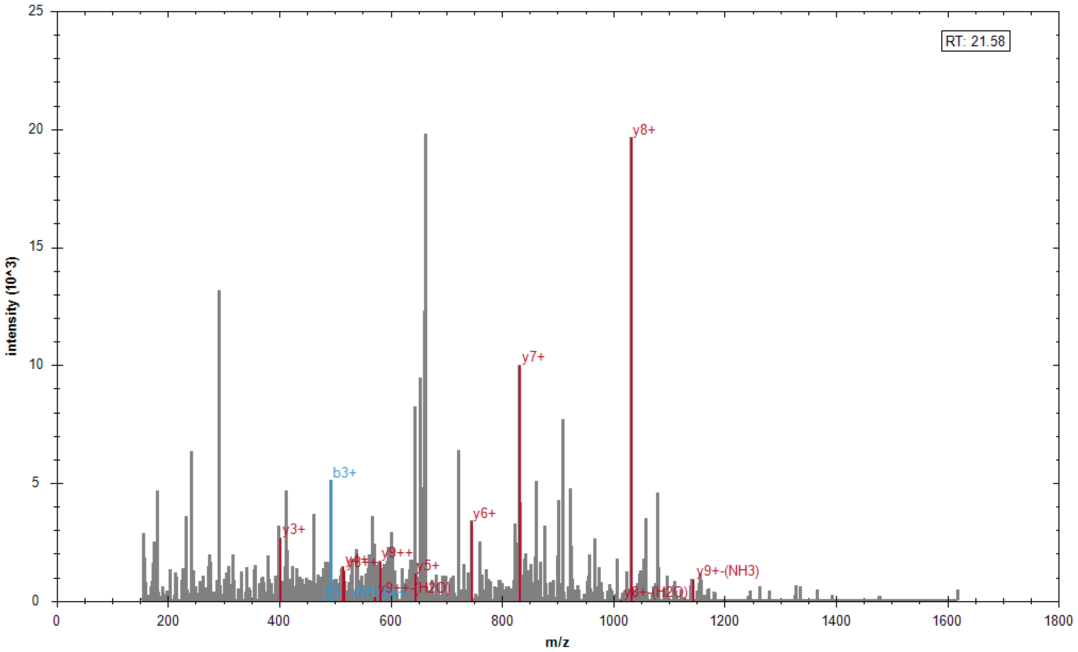

H1K33la

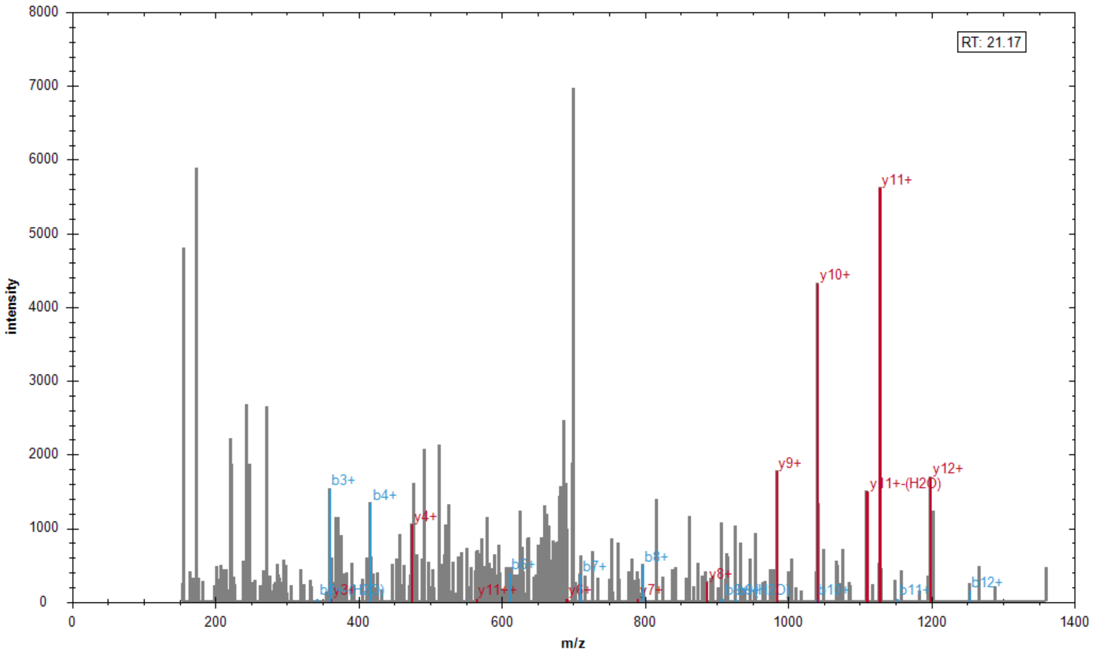

H3K23la

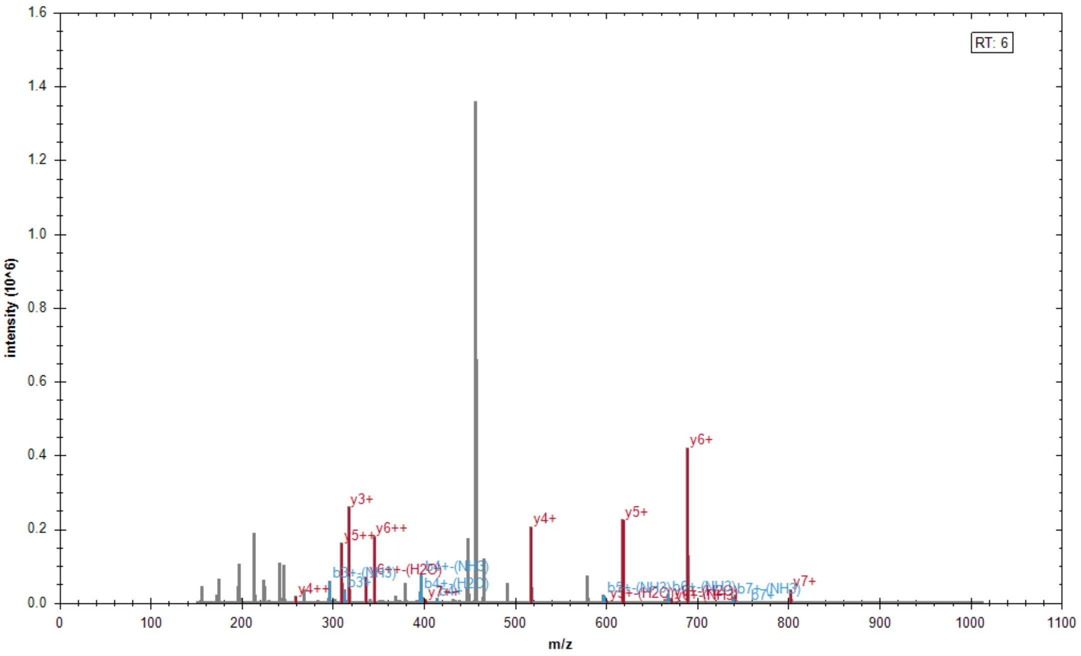

H3K18la

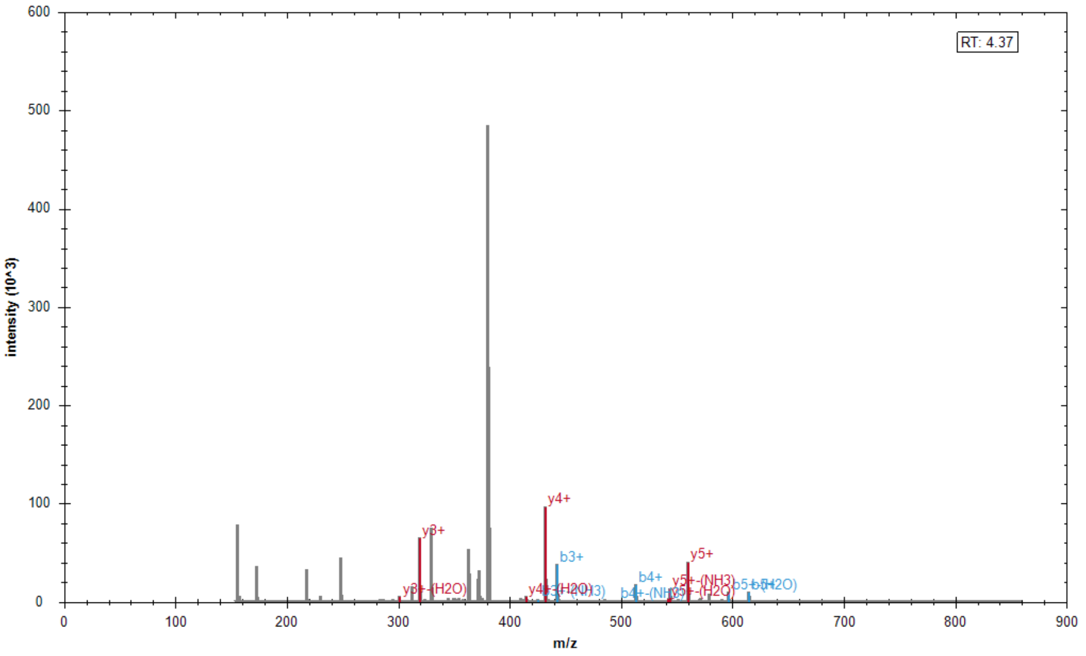

H4K8la

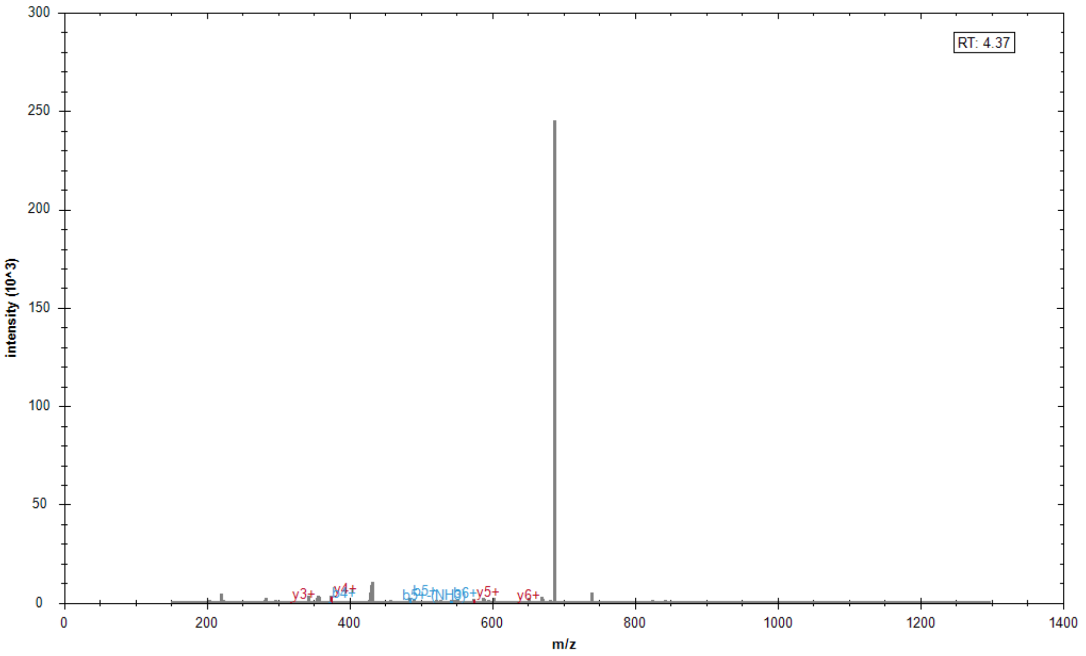

H4K5la

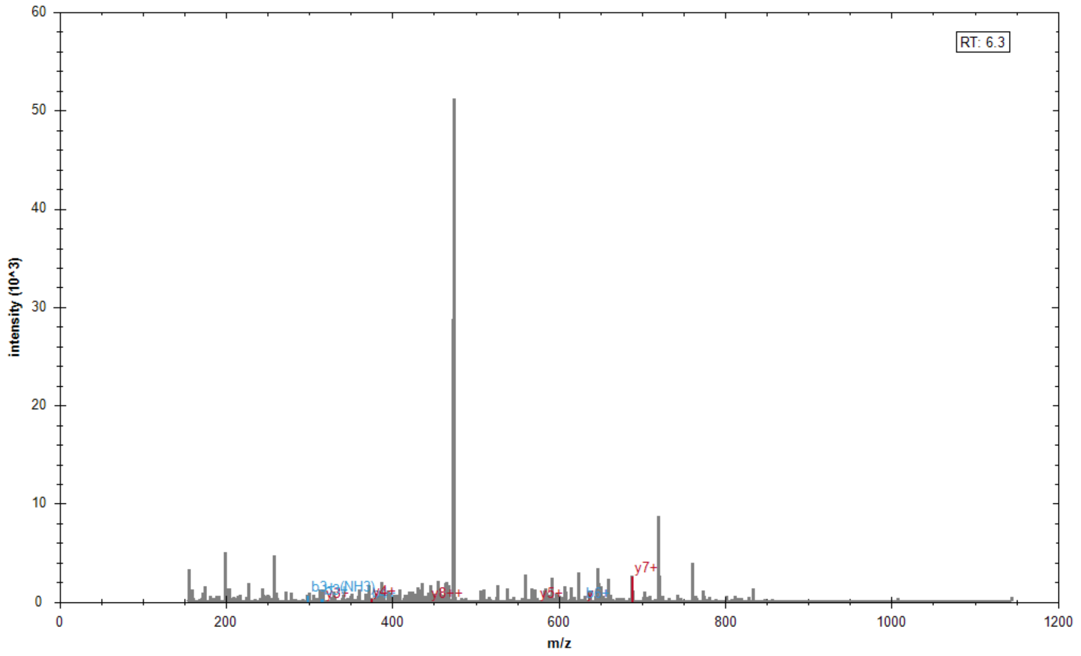

H2K5la

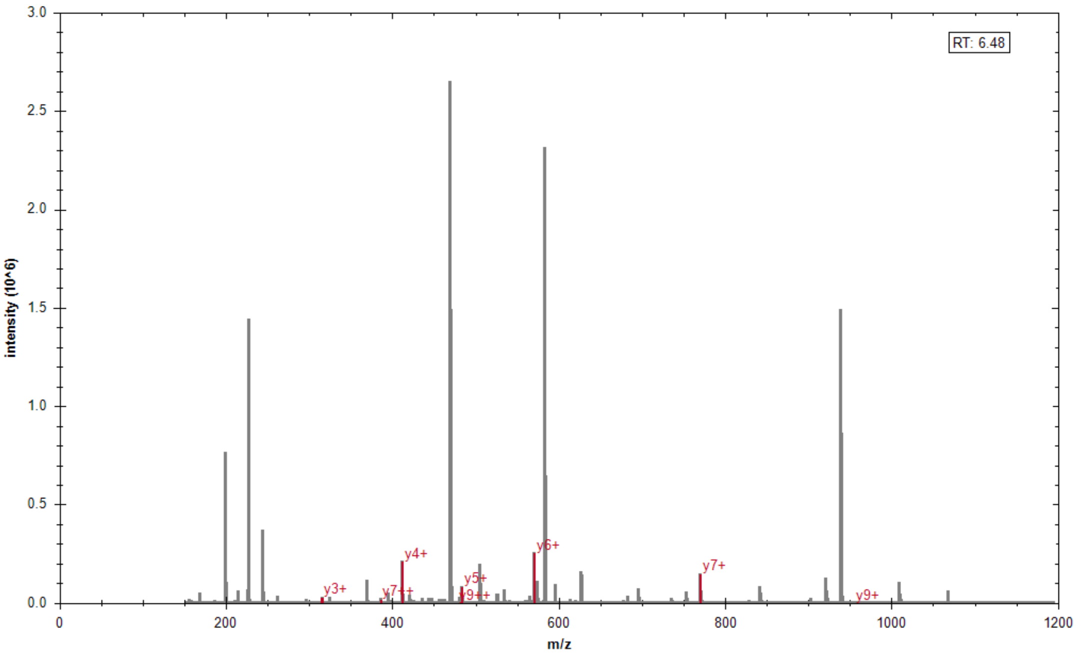

Supplement: Supplementary file 4 — Supplementary Materials [file 41419_2025_7962_MOESM4_ESM.pdf]
